# Inferring Gene-by-Environment Interactions with a Bayesian Whole-Genome Regression Model

**DOI:** 10.1016/j.ajhg.2020.08.009

**Published:** 2020-09-03

**Authors:** Matthew Kerin, Jonathan Marchini

**Affiliations:** 1 Wellcome Trust Center for Human Genetics, Oxford, OX3 7BN, UK; 2 Regeneron Genetics Center, Tarrytown, NY 10591, USA

**Keywords:** GxE interactions, GxE heritability, whole-genome regression, linear mixed model

## Abstract

The contribution of gene-by-environment (GxE) interactions for many human traits and diseases is poorly characterized. We propose a Bayesian whole-genome regression model for joint modeling of main genetic effects and GxE interactions in large-scale datasets, such as the UK Biobank, where many environmental variables have been measured. The method is called LEMMA (Linear Environment Mixed Model Analysis) and estimates a linear combination of environmental variables, called an environmental score (ES), that interacts with genetic markers throughout the genome. The ES provides a readily interpretable way to examine the combined effect of many environmental variables. The ES can be used both to estimate the proportion of phenotypic variance attributable to GxE effects and to test for GxE effects at genetic variants across the genome. GxE effects can induce heteroskedasticity in quantitative traits, and LEMMA accounts for this by using robust standard error estimates when testing for GxE effects. When applied to body mass index, systolic blood pressure, diastolic blood pressure, and pulse pressure in the UK Biobank, we estimate that 9.3%, 3.9%, 1.6%, and 12.5%, respectively, of phenotypic variance is explained by GxE interactions and that low-frequency variants explain most of this variance. We also identify three loci that interact with the estimated environmental scores ($-\mathrm{lo}g_{10}p>7.3$).

## Introduction

Despite long standing interest in gene-by-environment (GxE) interactions,^1^ this facet of genetic architecture remains poorly characterized in humans. Detection of GxE interactions is inherently more difficult than finding additive genetics in genome-wide association studies (GWASs). One difficulty is that of sample size: a commonly cited rule of thumb suggests that detection of interaction effects requires a sample size at least four times larger than that required to detect a main effect of comparable effect size.^2^ Another difficulty is that an individual’s environment, which evolves through time, is very hard to measure in a comprehensive way and is inherently high dimensional. Also, there are many environmental variables that could plausibly interact with the genome and many ways to combine them, and typically these factors are not all present in the same dataset. The recently released UK Biobank dataset, a large population cohort study with deep genotyping and sequencing and extensive phenotyping^3^ offers a unique opportunity for the exploration of GxE effects.^4^, ^5^, ^6^, ^7^, ^8^, ^9^, ^10^

It can be challenging to interpret statistical inference of interactions, which should not be interpreted as biological interaction.^11^^,^^12^ Specifically, the choice of scale for a quantitative phenotype can influence the extent to which interactions are detected.^13^ However, for discovery of associated loci, including interactions can increase power^14^ and explicitly modeling them genome wide, as we do in this paper, can be valuable in pointing the way to improving prediction models.

Models that consider environmental variables jointly can be advantageous, particularly if several environmental variables drive interactions at individual loci or if an unobserved environment driving interactions is better reflected by a combination of observed environments. StructLMM^7^ models the environmental similarity between individuals (over multiple environments) as a random effect and then tests each SNP independently for GxE interactions. However, StructLMM is not a whole-genome regression (WGR) model, so it does not account for the genome-wide contribution of all other variants, which is often a major component of phenotypic variance.

Advances in methods applied for detecting genetic main effects in standard GWASs have shown that linear mixed models (LMMs) can reduce false positive associations due to population structure and improve power by implicitly conditioning on other loci across the genome.^15^, ^16^, ^17^ Often these methods model the unobserved polygenic contribution as a multivariate Gaussian with covariance structure proportional to a genetic relationship matrix (GRM).^18^, ^19^, ^20^ This approach is mathematically equivalent to a WGR model with a Gaussian prior over SNP effect sizes.^15^ More flexible approaches that would allow for different prior distributions that better capture SNPs of small and large effects have been proposed in both the animal breeding^21^^,^^22^ and human literature.^23^, ^24^, ^25^ The BOLT-LMM method^17^ uses a mixture of Gaussians (MoG) prior and shows this can increase power for detecting associated loci in some (but not all) complex traits.

Here, we propose a method called Linear Environment Mixed Model Analysis (LEMMA), which aims to combine the advantages of WGR and modeling GxE with multiple environments and is applicable to large datasets with hundreds of thousands of individuals, such as the UK Biobank. Instead of assuming that the GxE effect over multiple environments is independent at each variant, as StructLMM does, we learn a single linear combination of environmental variables (which we call an environmental score [ES]) that has a common role in interaction effects genome wide. The ES is estimated within a Bayesian WGR model that uses two separate MoG priors on main genetic effects and GxE effects. We use variational inference to fit the model that is tractable for GxE analyses of biobank scale datasets with tens of environmental variables.

Estimating the ES satisfies one of the primary motivations of this work by providing a readily interpretable way to examine the combined effect of many environmental variables and how they might interact with genotype. A motivating example is the investigation of how modern obesogenic environments might accentuate the genetic risk of obesity. Tyrell et al.^26^ studied environments one at a time for their interaction with a body mass index (BMI) genetic risk score (GRS) and found several significant interactions. Our method allows joint analysis of environments that might plausibly better represent an obesogenic environment, negating the need to model each environment one at a time. Our other motivations when developing LEMMA were to develop a powerful method to detect GxE interactions and to estimate the proportion of variance that could be attributable to GxE interactions.

A LEMMA analysis has several distinct steps. First, the model is fitted with a large set of SNPs genome wide (e.g., all the SNPs that have been directly assayed on a genotyping chip). The estimated ES is then used to estimate the proportion of phenotypic variability that is explained by interactions with this ES (GxE heritability) via randomized Haseman-Elston (RHE) regression.^27^^,^^28^ This heritability analysis can be run on genotyped or imputed SNPs and can be stratified by minor allele frequency (MAF) and linkage disequilibrium (LD) for better interrogation of the genetic architecture of GxE interactions. The ES is also used for testing for GxE interactions one variant at a time, typically at a larger set of imputed SNPs in the dataset. We use “robust” standard errors when testing each variant for a GxE interaction, which helps control for the conditional heteroskedasticity caused by GxE interactions. We also suggest checks and solutions for the situation where environmental variables are themselves heritable and have a non-linear relationship to the trait of interest.

We compared LEMMA to existing approaches, such as StructLMM and F-tests, by using simulated data and applied the approach to UK Biobank data for BMI, systolic blood pressure (SBP), diastolic blood pressure (DBP), and pulse pressure (PP).

## Material and Methods

### Linear Environment Mixed Model Analysis (LEMMA)

The standard LMM used in genome wide association studies is written as

(Equation 1) y=Cα+u+ϵ,

where *y* is the centered and scaled N×1 vector of phenotypes, *C* is an N×L′ matrix of covariates with L′×1 fixed effects vector α, and *u* and ϵ are N×1 vectors of unobserved polygenic and residual effects vectors, respectively. Typically, *u* is modeled as a Gaussian with a mean of zero and covariance matrix $\sigma_{g}^{2}K$. Specification of the N×N kinship matrix *K* is an area of active research,^29^, ^30^, ^31^, ^32^ but the simplest approach is to let $K=XX^{T}/M$, where *X* is the N×M genotype matrix and columns of *X* (which usually correspond to SNPs) are normalized to have a mean of zero and variance one. This can equivalently be written as a Bayesian WGR model,

(Equation 2) y=Cα+Xβ+ϵ,

where

(Equation 3) $$\beta ∼N\left(0,\sigma_{g}^{2}/M\right).$$

Here β is an M×1 vector modeling the random effect of each SNP. This form corresponds to the so-called infinitesimal model where every SNP is allowed to have a small but non-zero effect on a given trait. To generalize the model to a non-infinitesimal genetic architecture, we model SNP effects with a mixture of Gaussian priors. This approach has been applied previously in human genetics^17^^,^^25^ and by the “Bayesian alphabet” of genomic prediction methods in the animal breeding literature.^21^^,^^22^^,^^33^

We extend this setup to model GxE interactions genome wide with a linear combination of multiple environmental variables by using

(Equation 4) Y=Cα+Xβ+Zγ+ϵ,

where

(Equation 5) Z=η⊙X,

(Equation 6) η=Ew,

(Equation 7) $$w∼N\left(0,I_{L}\right),$$

where *E* is an N×L matrix of environmental variables that could potentially be involved in GxE interactions and *w* is an L×1 vector of weights. Together they define the N×1 vector η, which is the linear combination of environments that we refer to as the ES. This ES is learned in tandem with SNP effects. We note that all environmental variables contained in *E* must also be contained in *C*, so L≤L′. We chose to model the interaction weights *w* with a Gaussian prior, but in theory, one could consider sparser priors, such as a spike and slab. We set the variance of the prior on *w* to the identity matrix $I_{L}$. Setting the prior variance of *w* to a parameter would be unidentifiable because any change in scale would be absorbed by the prior variances on the interaction effects γ (see $\sigma_{\gamma ,1}^{2}$ and $\sigma_{\gamma ,2}^{2}$ in Equations 8 and 9).

The N×M matrix *Z* contains all of the multiplicative interaction terms of the ES η with all of the genetic variants. We use the notation η⊙X for the element-wise product of η with each column of *X*. In other words, η⊙X=diag(η)X, where diag(η) is an N×N diagonal matrix with η as the diagonal. The vector of interaction effect sizes γ has dimension M×1.

We chose to use MoG priors on both the main genetic effects (β) and the interaction effects (γ) because this prior is very flexible and spans the range of genetic architectures from polygenic to a very sparse model. The priors are

(Equation 8) $$\beta_{j}|\sigma_{e}^{2},\lambda_{\beta },\sigma_{\beta ,1}^{2},\sigma_{\beta ,2}^{2}∼\lambda_{\beta }N\left(0,\sigma_{e}^{2}\sigma_{\beta ,1}^{2}\right)+\left(1-\lambda_{\beta }\right)N\left(0,\sigma_{e}^{2}\sigma_{\beta ,2}^{2}\right),$$

(Equation 9) $$\gamma_{j}|\sigma_{e}^{2},\lambda_{\gamma },\sigma_{\gamma ,1}^{2},\sigma_{\gamma ,2}^{2}∼\lambda_{\gamma }N\left(0,\sigma_{e}^{2}\sigma_{\gamma ,1}^{2}\right)+\left(1-\lambda_{\gamma }\right)N\left(0,\sigma_{e}^{2}\sigma_{\gamma ,2}^{2}\right).$$

We use standard Gaussian priors on the covariate and error terms.

(Equation 10) $$\alpha |\sigma_{\alpha }^{2}∼N\left(0,\sigma_{\alpha }^{2}\right)$$

(Equation 11) $$\epsilon |\sigma_{e}^{2}∼N\left(0,\sigma_{e}^{2}\right)$$

### Variational Inference

For notational convenience, we define θ={α,β,γ,w} as the set of latent variables, D:={X,E} the genetic and environmental data, and φ as the set of hyper parameters. Then the posterior p(θ|y,D,φ) is given by

(Equation 12) $$p\left(\theta |y,D,\varphi \right)\propto p\left(y|\theta ,D,\varphi \right)\prod_{c}p\left(\alpha_{c}|\varphi \right)\prod_{l}p\left(w_{l}\right)\prod_{j}p\left(\beta_{j},u_{j}|\varphi \right)\prod_{j}p\left(\gamma_{j},v_{j}|\varphi \right).$$

To evaluate the posterior, we use the variational inference framework, approximating the true posterior p(θ|y,D,φ) with a tractable alternative distribution q(θ;ν) governed by (variational) parameters ν. To make inference tractable, we use the standard mean-field assumption so that q(θ;ν) factorizes

(Equation 13) $$q\left(\theta ;\nu \right)=\prod_{c}q\left(\alpha_{c}\right)\prod_{l}q\left(w_{l}\right)\prod_{j}q\left(\beta_{j},u_{j}\right)\prod_{j}q\left(\gamma_{j},v_{j}\right).$$

To make q(θ;ν) a close approximation of the true posterior, we minimize the Kullback-Leibler (KL) divergence between q(θ;ν) and p(θ|y,D,φ) with respect to variational parameters ν. In this manner, the problem has been transformed from one of computing posterior distributions into one of optimization. We can show that minimizing the KL divergence is equivalent to maximizing a lower bound on the marginal log likelihood by observing

(Equation 14) $$KL\left(q∥p\right)=-E_{q}\left[\text{log}\frac{p\left(\theta |y,D,\varphi \right)}{q\left(\theta ;\nu \right)}\right],$$

(Equation 15) $$=-E_{q}\left[\text{log}\frac{p\left(\theta ,y|D,\varphi \right)}{q\left(\theta ;\nu \right)}\right]+E_{q}\left[\text{log}p\left(y|D,\varphi \right)\right],$$

(Equation 16) $$=-E_{q}\left[\text{log}\frac{p\left(\theta ,y|D,\varphi \right)}{q\left(\theta ;\nu \right)}\right]+\text{log}p\left(y|D,\varphi \right).$$

Thus, we can write

(Equation 17) $$F\left(\nu ;\varphi \right):=E_{q}\left[\text{log}\frac{p\left(\theta ,y|D,\varphi \right)}{q\left(\theta ;\nu \right)}\right]\leq \text{log}p\left(y|D,\varphi \right).$$

Here, F(ν;φ) is commonly referred to as the evidence lower bound (ELBO). As a result of the factorized form of Equation 13, we can cyclically update the approximate distribution for each latent variable in turn until we reach convergence.

Our model depends on a set of eight hyper-parameters $\varphi =\left\{\sigma_{e}^{2},{\left\{\sigma_{\beta ,i}^{2}\right\}}_{i=1}^{2},{\left\{\sigma_{\gamma ,i}^{2}\right\}}_{i=1}^{2},\lambda_{\beta },\lambda_{\gamma },\sigma_{\alpha }^{2}\right\}$. We set $\sigma_{\alpha }^{2}$ to a large constant to create a flat prior on the covariates, leaving seven unknowns. Similar methods have performed a grid search over hyper-parameter values (with either cross validation^17^ or the in-sample ELBO to identify the optimum^24^). For LEMMA, a grid search would be computationally demanding because the set of hyper-parameters is larger and we cannot efficiently perform multiple runs in parallel as done by Loh et al.^17^ Instead, we maximize a lower bound on the approximate log likelihood (the so-called ELBO) with respect to the hyper-parameters. In this manner, our approach can be viewed as a variational expectation maximization algorithm.^34^^,^^35^

Similar to the EM algorithm, the hyper-parameter maximization step can lead to slow exploration of the hyper-parameter space and thus to slow convergence of the LEMMA algorithm. We use an accelerator, SQUAREM,^36^ to speed up convergence. Given two estimates of the hyper-parameters $\varphi_{t-2}$ and $\varphi_{t-1}$, we can adjust the maximized estimate $\varphi_{t}$ with

(Equation 18) $${\tilde{\varphi }}_{t}\left(v_{t}\right)=\varphi_{t-2}-2v_{t}\Delta \varphi_{t-1}+v_{t}^{2}\Delta^{2}\varphi_{t},$$

where $\Delta \varphi_{t-1}=\varphi_{t-1}-\varphi_{t-2}$ and $\Delta^{2}\varphi_{t}=\varphi_{t}-2\varphi_{t-1}+\varphi_{t-2}$. Thus, the new adjusted estimate ${\tilde{\varphi }}_{t}\left(v_{t}\right)$ is a continuous function of the step size $v_{t}$, which yields the original estimate $\varphi_{t}$ for $v_{t}=-1$. As recommended by Varadhan et al.,^36^ we set $v_{t}=\min\left(-1,-{\left\|\Delta \varphi_{t-1}\right\|}_{2}^{2}/{\;\|\Delta^{2}\varphi_{t}\|}_{2}^{2}\right)$. Occasionally this yields an estimate that is either outside of the domain of φ or leads to a state with a worse ELBO than the previous state. For the first issue, we use a simple backtracking method of halving the distance between $v_{t}$ and −1, and for the second, we simply judge model convergence when the absolute change in the ELBO drops below a given threshold. We use the same convergence criterion as the BOLT-LMM method:^17^ namely that a full pass through all latent variables yields an absolute change of less than 0.01 in the approximate log likelihood (ELBO). Figure S17 shows the evolution of the ES parameter estimates for the four UK Biobank traits we analyzed and illustrates that, at the point of convergence, the parameters appear stable.

### Identifying GxE-Associated Loci

After convergence of the LEMMA variational inference algorithm, we obtain posterior mean estimates of $\hat{\beta },\hat{\gamma }$, and $\hat{\eta }=E\hat{w}$. From these, we construct residualized phenotypes by following a leave-one-chromosome-out (LOCO) scheme:

(Equation 19) $$y_{\text{resid}-\text{LOCO}}=y-C\hat{\alpha }-X_{\text{LOCO}}{\hat{\beta }}_{\text{LOCO}}-\hat{\eta }⊙X_{\text{LOCO}}{\hat{\gamma }}_{\text{LOCO}}.$$

$X_{\mathrm{LOCO}}$ denotes the genotype matrix excluding SNPs on the same chromosome of the test SNP, and $\beta_{\text{LOCO}}$ and $\gamma_{\text{LOCO}}$ are constructed similarly. Using a LOCO scheme has been shown to increase power in LMMs because the effect of the test SNP is conditioned on the effects on a large proportion of the rest of the genome.^16^^,^^19^

For each imputed SNP, we then perform hypothesis tests $\beta_{\text{test}}\neq 0$ and $\gamma_{\text{test}}\neq 0$ by using the linear model

(Equation 20) $$y_{\text{resid}-\text{LOCO}}=x_{\text{test}}\beta_{\text{test}}+\left(\hat{\eta }⊙x_{\text{test}}\right)\gamma_{\text{test}}+\epsilon ,$$

(Equation 21) =Hτ+ϵ.

Here, *H* is the N×2 design matrix with first and second columns containing $x_{\text{test}}$ and $\hat{\eta }⊙x_{\text{test}}$, respectively, and τ is the 2×1 vector containing parameters $\beta_{\text{test}}$ and $\gamma_{\text{test}}$, which are the main genetic effect and interaction effect of the SNP being tested.

Assuming that ϵ has a mean of zero and covariance matrix Ω, we can use the standard ordinary least squares (OLS) estimator

(Equation 22) $$\hat{\tau }={\left(H^{T}H\right)}^{-1}H^{T}y,$$

which (under certain regularity conditions) is asymptotically normally distributed with mean τ and variance $\mathrm{Var}\left(\hat{\tau }\right)$. By assuming the residual phenotype is homoskedastic, that is that $\Omega ={\hat{\sigma }}_{e}^{2}I$, we can obtain the usual variance estimator given by

(Equation 23) $$\mathrm{Var}\left(\hat{\tau }\right)={\hat{\sigma }}_{e}^{2}{\left(H^{T}H\right)}^{-1}.$$

It has previously been observed that GxE interaction tests are likely to suffer from conditional heteroskedasticity,^37^ and hence, the homoskedastic variance estimator is likely to underestimate the true variance.^38^ We explain this phenomenon in detail in the Supplemental Notes.

To overcome this, we use robust standard errors, alternatively called Huber-White, sandwich, or “heteroskedastic consistent” errors,^39^^,^^40^ that are standard tools in economics^41^ and have previously been proposed for use in GxE interaction studies.^37^^,^^42^^,^^43^ We further include a small adjustment that reduces bias in small samples.^44^ This yields the variance estimator

(Equation 24) $$\mathrm{Var}\left(\hat{\tau }\right)={\left(H^{T}H\right)}^{-1}H^{T}\hat{\Sigma }H{\left(H^{T}H\right)}^{-1},$$

where $\hat{\Sigma }$ is a diagonal matrix with ${\hat{\Sigma }}_{ii}=\left({\hat{\epsilon }}_{i}^{2}/{\left(1-h_{ii}\right)}^{2}\right)$, where $\hat{\epsilon }=y-H\hat{\tau }$ and $h=H{\left(H^{T}H\right)}^{-1}H^{T}$. Hence, our GxE test statistic is given by

(Equation 25) $$\frac{{\hat{\gamma }}_{\text{test}}^{2}}{\mathrm{Var}\left({\hat{\gamma }}_{\text{test}}\right)}$$

and, under the null hypothesis, is asymptotically distributed as $\chi_{1}^{2}$. Because main effects tests are not sensitive to assumptions of heteroskedasticity in the same way that GxE tests are,^37^ we use a simple t test to test the hypothesis $\beta_{\text{test}}\neq 0$.

### Heritability Estimation

Previous GWR methods^24^^,^^45^^,^^46^ have shown that it is possible to rearrange the model hyper-parameters to gain an estimate of trait heritability. We find that in our variational framework, this approach underestimates trait heritability because of the tendency of mean-field variational inference to underestimate the posterior variance of each parameter. Instead, we treat the posterior mean ${\hat{\eta }}_{\mathrm{LEMMA}}$ as a fixed effect and use RHE regression^27^^,^^28^^,^^47^ to estimate heritability with a single SNP component^27^ (RHE-SC) and multiple SNP components^28^ (RHE-LDMS). With the multi-component model, SNPs are stratified into a total of 20 bins: 5 MAF bins (≤0.1, 0.1< MAF ≤0.2, 0.2< MAF ≤0.3, 0.3< MAF ≤0.4, and 0.4< MAF ≤0.5) and 4 LD score quantiles.

The single component model is given by

(Equation 26) $$y∼N\left(E\alpha ,\sigma_{\beta }^{2}K+\sigma_{\gamma }^{2}\hat{V}+\sigma_{e}^{2}I\right),$$

where $K=XX^{T}/M$, $\hat{V}=Z\left(\hat{\eta }\right)Z{\left(\hat{\eta }\right)}^{T}/M$, and $Z\left(\hat{\eta }\right)=\mathrm{diag}\left(\hat{\eta }\right)X$. HE regression is a method of moments estimator that fits the variance components $\left(\sigma_{\beta }^{2},\sigma_{\gamma }^{2},\sigma_{e}^{2}\right)$ to minimize the difference between the empirical and expected covariances. This is mathematically equivalent to solving the following linear system:

(Equation 27) $$\left(\begin{array}{ccc} \text{tr}\left(K^{2}\right) & \text{tr}\left(KV\right) & \text{tr}\left(K\right)\\ \text{tr}\left(KV\right) & \text{tr}\left(V^{2}\right) & \text{tr}\left(K\right)\\ \text{tr}\left(K\right) & \text{tr}\left(V\right) & N \end{array}\right)\left(\begin{array}{c} \sigma_{\beta }^{2}\\ \sigma_{\gamma }^{2}\\ \sigma_{e}^{2} \end{array}\right)=\left(\begin{array}{c} y^{T}Ky\\ y^{T}Vy\\ y^{T}y \end{array}\right).$$

Wu et al.^27^ showed that this system can be solved in O(NMB) time (for small *B*) without ever forming the kinship matrices *K* and *V* with Hutchinson’s estimator and that covariates can be efficiently projected out of the phenotype, genotypes, and interaction matrix *Z* with minimal additional cost. Pazokitoroudi et al.^28^ give an extension to multiple components and show that variance estimates can be obtained with the block jackknife.

Speed et al.^48^ show that the usual form for $h_{G}^{2}$, the proportion of trait variance explained by additive genetic effects, given by

(Equation 28) $${\hat{h}}_{G}^{2}=\frac{{\hat{\sigma }}_{\beta }^{2}}{{\hat{\sigma }}_{\beta }^{2}+{\hat{\sigma }}_{\gamma }^{2}+{\hat{\sigma }}_{e}^{2}},$$

holds only when genotype matrix *X* is standardized to have a column mean of zero and column variance of one. Although this is true in expectation for $\hat{Z}$ (assuming that $\mathrm{Cov}\left(\hat{\eta },X_{j}\right)=0,\;\forall j\in \left\{1,M\right\}$), this is not guaranteed. To obtain a column mean of zero, we include an intercept of ones among the covariates that are projected out of the phenotype, genotypes, and interaction matrix. To account for columns’ having variance not equal to one, we use a more general form of the heritability estimator (see Speed et al.^48^ for details)

(Equation 29) $${\hat{h}}_{\text{GxE}}^{2}=\frac{{\hat{\sigma }}_{\gamma }^{2}\text{tr}\left(\hat{V}\right)/N}{\sigma_{\beta }^{2}+{\hat{\sigma }}_{\gamma }^{2}\text{tr}\left(\hat{V}\right)/N+{\hat{\sigma }}_{e}^{2}}.$$

### Computational Efficiency

We implement a number of steps to improve computational and memory efficiency, including vectorization using SIMD extensions, compressed data formats, pre-computing quantities, parallel computing with OpenMPI, and the use of the well-optimized Intel Math Kernel Library. Full details are given in the Supplemental Notes.

### Detecting Squared Environmental Dependence

By default, each of the *L* environmental variables is tested against the phenotype for significant squared effects. To do this, LEMMA tests the hypothesis $\beta_{l}\neq 0$ by using the following linear model:

(Equation 30) $$y=1\alpha_{0}+C\alpha +E_{l}^{2}\beta_{l}+\epsilon .$$

The squared effect of any environmental variables with a p value less than 0.01 (Bonferroni correction for *L* multiple tests) are added to the matrix of covariates *C*.

### Controlling for Covariates

Unlike in BOLT-LMM,^17^ it is not possible to efficiently project covariates out of the model (y,X,Z) because the multiplicative interaction matrix *Z* changes after each pass through the data. Instead, the LEMMA software package can either regress covariates out of the phenotype or model the covariates as random effects in the variational framework. For our analyses of the UK Biobank, we included all covariates within the variational model.

### Comparison to Existing GxE Methods

We compare LEMMA to three other single SNP methods that jointly model interactions with multiple environments. The first comparison method, StructLMM,^7^ is a method that uses a random effects term, *u*, to model environmental similarity instead of genetic similarity. Specifically, StructLMM uses the model

(Equation 31) $$y∼N\left(C\alpha +x_{\text{test}}\beta ,\sigma_{\text{GxE}}^{2}\mathrm{diag}\left(x_{\text{test}}\right)\Sigma \mathrm{diag}\left(x_{\text{test}}\right)+\sigma_{e}^{2}\Sigma +\sigma_{n}^{2}I\right)$$

to test the hypothesis $\sigma_{\text{GxE}}^{2}\neq 0$. Here, *C* is the matrix of covariates with fixed effects α, $x_{\text{test}}$ is the focal variant, and $\Sigma =EE^{T}$ is the environmental similarity matrix (where *E* is an N×L matrix of environmental variables). Although StructLMM provides both an interaction test and a joint test that looks for non-zero main and interaction effects at each SNP, we use only the interaction test in our comparisons. Finally, we note that StructLMM recommends “gaussianizing” the phenotype as a pre-processing step; however, we just center and scale the phenotype for consistency with our other methods.

Our second and third comparison methods use equivalent information to StructLMM in a fixed effects framework. Consider the linear model

(Equation 32) $$y=C\alpha +E\alpha \prime +x_{\text{test}}\beta_{\text{test}}+x_{\text{test}}⊙E\gamma +\epsilon$$

(Equation 33) =Hτ+ϵ,

where *H* is formed from column-wise concatenation of $\left[C,E,x_{\text{test}},\mathrm{diag}\left(x_{\text{test}}\right)E\right]$ and τ is the corresponding vector of fixed effects. Let *R* be the indicator matrix such that Rτ=γ. We wish to test the null hypothesis $H_{0}:\gamma =0$. Assuming that ϵ has a mean of zero and covariance matrix Ω, we can use the standard OLS estimator $\hat{\tau }={\left(H^{T}H\right)}^{-1}H^{T}y$, which (under certain regularity conditions) is asymptotically distributed as normal with mean τ and variance given by $\mathrm{Var}\left(\hat{\tau }\right)={\left(H^{T}H\right)}^{-1}H^{T}\Omega H{\left(H^{T}H\right)}^{-1}.$ Assuming homoskedasticity yields the standard F test statistic,

(Equation 34) $$F_{\text{test}}=\frac{{\left(R\hat{\tau }\right)}^{T}{\left(R{\left(H^{T}H\right)}^{-1}R^{T}\right)}^{-1}\left(R\hat{\tau }\right)/L}{{\hat{\sigma }}_{e}^{2}},$$

which follows an $F_{d_{1}-d_{0},N-d_{1}}$ distribution under the null hypothesis, where $d_{1}$ is the column rank of *H* and $d_{0}$ is the column rank of *H* under the null hypothesis. Alternatively, we can use the same robust standard error used in the LEMMA test statistic

(Equation 35) $$F_{\text{robust}}={\left(R\hat{\tau }\right)}^{T}{\left(R{\left(H^{T}H\right)}^{-1}H^{T}\hat{\Omega }H{\left(H^{T}H\right)}^{-1}R^{T}\right)}^{-1}\left(R\hat{\tau }\right),$$

where $\hat{\Omega }$ is a diagonal matrix with ${\hat{\Omega }}_{ii}=\frac{{\hat{\epsilon }}_{i}^{2}}{{\left(1-h_{ii}\right)}^{2}}$, $\hat{\epsilon }=y-H\hat{\tau }$ and $h=H{\left(H^{T}H\right)}^{-1}H$. Then $F_{\mathrm{robust}}$ is asymptotically distributed as $\chi_{d_{3}}^{2}$, where $d_{3}$ is the rank of $HR^{T}$. In our simulations, we refer to this as the robust F-test.

### SNP-Specific Interaction Profile

The SNP-specific interaction profile is defined as $\eta_{LS}=Ew_{LS}$, where $w_{LS}$ is the least-squares parameter estimate of *w* in the single SNP model

(Equation 36) $$y=C\alpha +x_{\mathrm{test}}\beta_{\mathrm{test}}+x_{\mathrm{test}}⊙Ew+\epsilon$$

and y,C, and *E* are the data matrices defined below. The correlation between $\eta_{LS}$ for a given SNP and the ES estimated by LEMMA can be viewed as a proxy for how well LEMMA captures the GxE interactions at that locus.

### UK Biobank Analysis

We used real genotype and phenotype data from the UK Biobank, which is a large prospective cohort study of approximately 500,000 individuals living in the UK.^3^ To account for potential confounding effects of population structure, we first subset down to the white British subset of 344,068 individuals used by Bycroft et al.^3^ in a GWAS on human height. This represents unrelated individuals who self-report white British ethnicity and whose genetic data projected onto principal components lies within the white British cluster.^3^ After sub-setting down to individuals who had complete data across the phenotype, covariates, and environmental factors (see below), we were left with approximately 280,000 samples per trait (Table S1). Finally, we filtered genetic data on the basis of MAF (≥0.01) and IMPUTE-info score (≥0.3), leaving approximately 642,000 genotyped variants (Table S1) and 10,295,038 imputed variants per trait. For each trait, we included ${\text{age}}^{3}$, ${\text{age}}^{2}\times$ gender, ${\text{age}}^{3}\times$ gender, a binary indicator for the genotype chip, and the top 20 genetic principal components as additional covariates.

BMI was derived from height and weight measurements made during the first assessment visit (instance “0” of field 21001), and readings more than six standard deviations from the population mean were set to missing. logBMI and INT(BMI) refer to BMI after applying a log transformation and an inverse normal transformation (applied separately to males and females), respectively.

After calculating the mean SBP and DBP by using automated blood pressure readings from the first assessment visit (fields 4080 and 4079), we adjusted for medication usage by adding 15 mmHg and 10 mmHg to SBP and DBP, respectively.^49^ Data from manual measurements (fields 93 and 94) were used in the rare instance that no automated reading was available. Blood pressure readings more than four standard deviations from the mean were set to missing. PP was then calculated as SBP minus DBP.

For our GxE analyses, we made use of 42 environmental variables from the UK Biobank, similar to those used in previous GxE analyses of BMI in the UK Biobank.^7^^,^^50^ From the data provided by the UK Biobank, we included seven continuous environmental variables (“age when attended assessment centre,” “sleep duration,” “time spent watching television,” “number of days/week walked 10+ minutes,” “number of days/week of moderate physical activity 10+ minutes,” “number of days/week of vigorous physical activity 10+ minutes,” and “Townsend deprivation index at recruitment”), one ordinal environmental variable (“alcohol intake frequency”), nine dietary ordinal variables (“salt added to food,” “oily fish intake,” “non-oily fish intake,” “processed meat intake,” “poultry intake,” “beef intake,” “lamb intake,” “pork intake,” and “cheese intake”) and two dietary continuous variables (“tea intake” and “cooked vegetable intake”). We further derived one categorical variable (“is current smoker” from the responses given in the UK Biobank field “smoking status”) and one continuous variable (“sleep sd,” the number of standard deviations from the population mean sleep duration). For analyses of blood pressure, we additionally included one further continuous variable, “waist circumference.” This left 11 dietary variables and ten non-dietary variables (11 for blood pressure traits). In addition, we included multiplicative interactions between participants’ age and gender with all non-dietary variables and included the main effect of gender, giving the data matrix *E* a total of 42 columns (45 for blood pressure traits). Before running LEMMA, each column was standardized as

(Equation 37) $$E_{ij}=\frac{E_{ij}-\text{mean}\left(E_{:,j}\right)}{\text{sd}\left(E_{:,j}\right)}.$$

In all cases where participants responded with “prefer not to answer,” “do not know,” or “none of the above,” we set the value to missing. For three continuous variables (“time spent watching television,” “tea intake,” and “cooked vegetable intake”), we removed the 99^th^ percentile, and for “sleep duration,” we removed both the 1^st^ and 99^th^ percentiles.

After running LEMMA, we found it convenient to interpret weights corresponding to a re-scaled data matrix $E_{1}$. Assuming the column space of *E* and $E_{1}$ is the same, weights $w_{1}$ that correspond to $E_{1}$ can be extracted from the ES via least-squares

(Equation 38) $$w_{1}={\left(E_{1}^{T}E_{1}\right)}^{-1}E_{1}^{T}{\hat{\eta }}_{\text{LEMMA}},$$

where ${\hat{\eta }}_{\mathrm{LEMMA}}$ represents the ES. We note that, although multivariate linear regression is invariant to a re-scaling of the design matrix, ridge regression is not because of the penalization place on the magnitude of the learned parameters. However, because the magnitude of the weights from our UK Biobank analysis is typically small (less than 0.2) compared to the standard deviation of our Gaussian prior (1), in this case, the re-scaling makes minimal difference.

Re-coded data matrix $E_{1}$ was formed with one column for each of the 11 dietary variables (normalized to have a mean of zero and variance of one) and three columns for each of the ten (11 for blood pressure traits) non-dietary variables; the first column was augmented by a binary male indicator vector, the second by a binary female indicator vector, and the third by a continuous vector of participant age. Columns augmented by male and female binary indicator vectors were normalized to have a mean of zero and variance one (not including zeros due to augmentation), apart from age (scaled to represent the number of decades aged past 40 years). Columns augmented by age were normalized first and then multiplied by age on the per-decade scale. We further included indicator columns for men and women, which can be interpreted as gender-specific intercepts and is equivalent to including an intercept and a binary column for only one gender (men or women). We note this leaves 43 (46) columns where the extra column comes from including an intercept within the column space of $E_{1}$ and is necessary because some columns have a mean not equal to zero. Thus, the column space of $E_{1}$ is equivalent to *E* under the constraint that the ES has a mean of zero.

### Simulation Studies

Genetic data was sub-sampled from the UK Biobank by default with N=25,000 unrelated individuals of mixed ancestry and M=100,000 genotyped SNPs. Environmental variables were simulated from a standard Gaussian distribution. By default, we constructed phenotypes with 2,500 causal main effects and 1,250 causal interaction effects explaining 20% and 5% of trait variance, respectively. For each phenotype, we constructed a weighted average of the environmental variables, which we used to simulate multiplicative interaction effects. Environments with a non-zero weight are referred to as active. All non-zero effects were drawn from SNPs in the first half of each chromosome, allowing us to test the calibration of each method on “null” SNPs from the second half of each chromosome. To allow for direct power comparisons across different scenarios, we included an additional 60 SNPs with standardized effect sizes that together accounted for 1% of trait variance with their main effects and 1% of trait variance with their interaction effects. Finally, a further 1% of trait variance was modeled via the first genetic principal component (PC). For all methods, we included the first genetic PC as a covariate. For each method, we calculated power as the proportion of the SNPs of standardized effect identified at a threshold of p<0.01.

In simulations used to test RHE regression, phenotypes were constructed with 10,000 causal main effects, explaining 20% of trait variance, and in simulations with non-zero GxE heritability, with 10,000 causal SNPs with interaction effects.

### Model Misspecification

We simulated a scenario where a disease trait *Y* depends non-linearly on a heritable environmental factor *S*. More explicitly, suppose that *X* is the centered and scaled genotype matrix so that columns have a mean of zero and variance of one, that *S* is modeled as

(Equation 39) $$S=X\tau +\epsilon_{s},$$

where $\epsilon_{s}∼N\left(0,\left(1-h_{\tau }^{2}\right)I\right)$, τ models random SNP effects for *S*, and trait *Y* is given by

(Equation 40) $$Y|a=aS^{2}+X\beta +\epsilon .$$

Here, *a* is a constant that we use to control the strength of the contribution of $S^{2}$ to *Y*, $\epsilon ∼N\left(0,\left(1-h_{\beta }^{2}\right)\right)$ and β is the random SNP effects for *Y*. For simulation, we suppose that τ and β have spike and slab priors

(Equation 41) $$\tau_{j}|v_{j}∼v_{j}N\left(0,\frac{h_{\tau }^{2}}{P\lambda_{\tau }}\right)+\left(1-v_{j}\right)\delta_{0}\left(\tau_{j}\right),$$

(Equation 42) $$\beta_{j}|u_{j}∼u_{j}N\left(0,\frac{h_{\beta }^{2}}{P\lambda_{\beta }}\right)+\left(1-u_{j}\right)\delta_{0}\left(\beta_{j}\right),$$

(Equation 43) $$v_{j}∼\text{Ber}\left(\lambda_{\tau }\right),$$

(Equation 44) $$u_{j}∼\text{ Ber}\left(\lambda_{\beta }\right).$$

## Results

### Performance on Simulated Data

**Figure 1:**
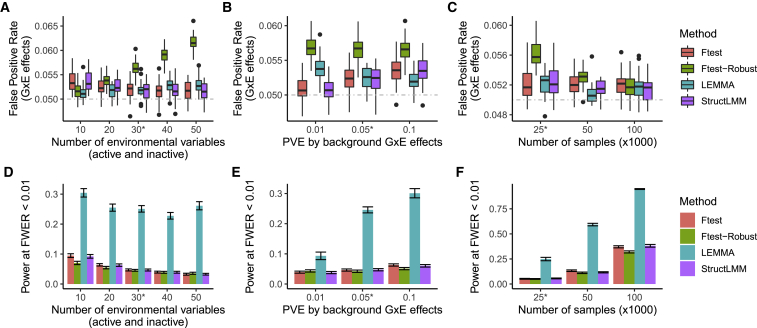
Type I Error and Power of Tests to Detect GxE Effects in Simulation (A–C) Comparison of false positive rate as the number of environments increases (A), as phenotype variance explained by GxE effects increases (B), or as the number of samples increases (C). (D–F) Analogous comparison of the power to detect GxE interactions. Simulations used genotypes subsampled from the UK Biobank and by default contained N=25,000 samples, M=100,000 SNPs, six environmental variables that contributed to the ES, and 24 that did not (default parameters denoted by stars). Error bars extend to mean +/− 1× standard error of the mean. We assess power (at family-wise error rate [FWER] <0.01) to detect 60 causal SNPs whose GxE effect each explained 0.00016% of trait variance. See Materials and Methods for full details of phenotype construction.

Figure 1 compares the ability of different methods to detect GxE interactions at SNPs in simulations where a single true ES interacts with SNPs across the genome. Figure S1 shows the false positive rate (FPR) to detect main effects. We compared our default version of LEMMA, which uses robust standard errors, StructLMM, a simple F-test of interaction, and an F-test that uses robust standard errors (see Material and Methods). The simulations vary GxE heritability, the total number of environmental variables, and sample size. When sample size is large (N = 100,000), all the methods have reasonable control of FPR and LEMMA controls FPR at least as well as other methods across the range of simulations. When sample size is smaller (N = 25,000), the robust F-test performs less well as the number of environments grows (Figure 1A) and the F-test and StructLMM perform less well as the amount of GxE variance increases (Figure 1B). When we increase the sample size to N = 200,000, we still find that LEMMA has a slighty inflated type I error rate (see Figure S2).

It is interesting that all the methods we tested have a slightly inflated type I error, and this is most likely due to a number of different reasons. StructLMM and the F-test fit a model at each variant and ignore GxE effects at other loci, which can induce heteroskedasticity that can inflate type I error.^37^^,^^43^ We used robust standard errors for the robust F-test, but it seems that this approximation works best when the number of environmental traits is small. LEMMA does account for GxE effects at other loci and also uses robust standard errors, but it still has a slightly inflated type I error that gets worse as the number of environments increases (Figures 1A and S2). In parallel simulations (see Figure S3), we find that our model slightly over-estimates GxE heritability as the number of environments increases. Because our simulations test for GxE effects at SNPs used to estimate the ES, we suspect that the type I error inflation is due to this two-stage approach.

When there is a single true ES involved in GxE interactions, we found that LEMMA provided a substantial power increase (Figures 1 and S4). StructLMM and F-tests have very similar power in these simulations, although previous work suggests that StructLMM may outperform the F-test in small samples.^7^

When estimating the GxE heritability of the LEMMA ES by using RHE regression with a single SNP component (RHE-SC), we observed some upward bias as the number of environments increases. This effect is ameliorated by increasing sample size (see Figure S3), suggesting that the influence of over-fitting in our Biobank analyses is mild. In 20 simulations with L=30 environmental variables, N=100,000 samples, and true GxE heritability of 5%, we observed a mean GxE heritability of 5.2%. Figure S5 further illustrates the ES estimation accuracy of LEMMA.

Finally, we ran LEMMA on two sets of simulated datasets (N = 25,000) with causal SNPs chosen either randomly or to be low frequency (MAF< 0.1). We used the ES estimated from each simulated dataset to estimate $h_{G}^{2}$ and $h_{\mathrm{GxE}}^{2}$ by using RHE with SNPs stratified by MAF and LD (RHE-LDMS) and then without any stratification (RHE-SC). Previous studies have established that estimating heritability with a single SNP component makes assumptions about the relationship between MAF, LD, and trait architecture that may not hold up in practice,^48^^,^^51^ whereas stratifying SNPs into bins according to MAF and LDscore (the LDMS approach) is relatively unbiased.^51^, ^52^, ^53^ Figure S6 confirms that stratifying by MAF and LD results in accurate heritability estimates irrespective of the MAF distribution of causal SNPs and suggests that this method can be used to interrogate the MAF distribution of GxE components of a trait via LEMMA. However, when causal SNPs are low frequency, not stratifying by MAF and LD results in underestimation of $h_{G}^{2}$.

### Controlling for Heritable Environmental Variables

Previous work by Tchetgen et al.^42^ has shown that misspecifying the functional form of an environmental variable can induce heteroskedasticity into tests for GxE interactions. The authors further show that use of robust standard errors will control for this heteroskedasticity but only if the environment is independent of the variant being tested. Independence between genotypes and the misspecified environment is important because it means that the (least-squares) mean estimator is still unbiased.

**Figure 2:**
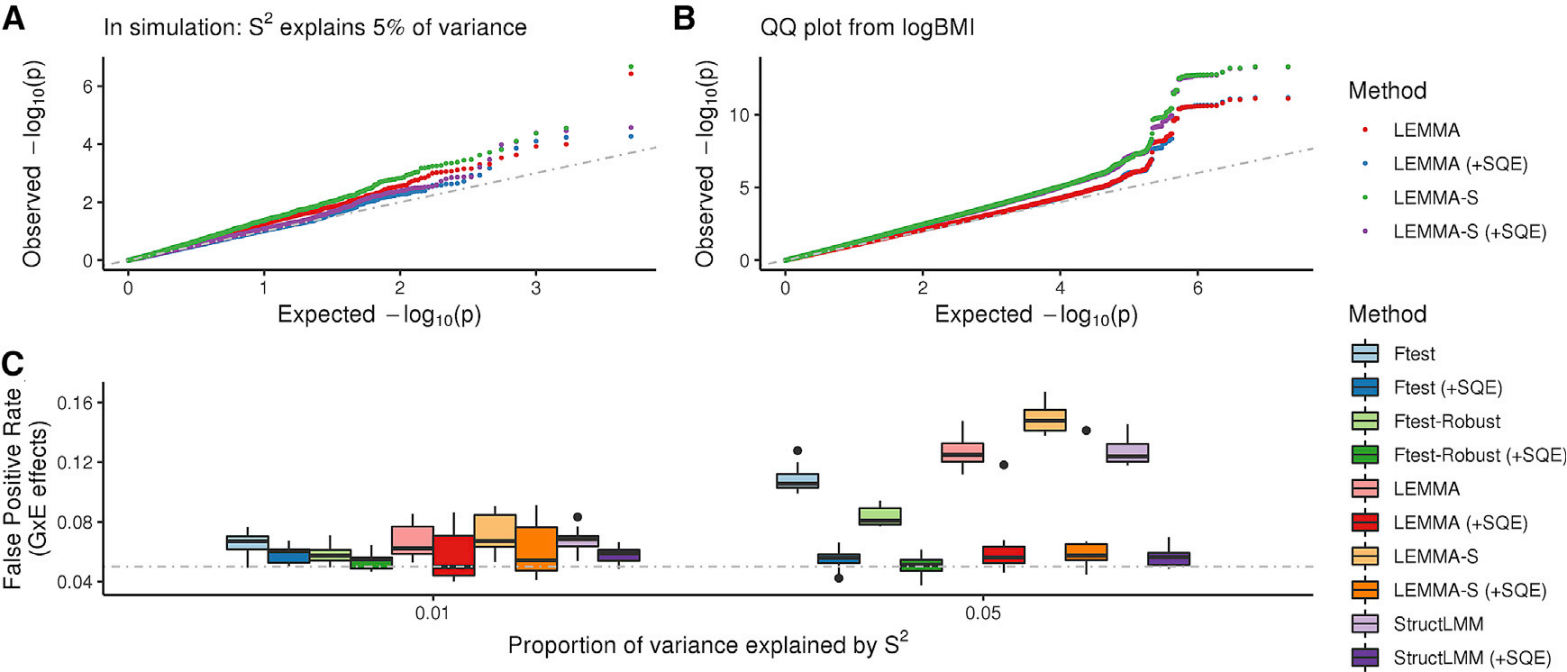
Bias from Model Misspecification of a Heritable Environmental Variable (A) Comparison of GxE association test statistics from a single simulation where non-linear dependence on the confounder explains 5% of trait variance. FPR at heritable sites of the misspecified environment only. (B) Comparison of GxE association test statistics from an analysis of logBMI in 281,149 participants from the UK Biobank. (C) FPR at heritable sites of the misspecified environment while the strength of squared dependence varies. 20 repeats per scenario. Abbreviations are as follows: LEMMA-S, LEMMA with non-robust variances used to compute test statistics; (+SQE), significant squared environmental variables (Bonferroni correction) included as additional covariates.

However, environmental variables themselves often have a genetic basis. We therefore performed simulations where the phenotype depended on the non-linear (squared) effect of a heritable environmental variable. In simulation (Figures 2A and 2C), we observed that misspecification of the environmental variable can cause substantial inflation in GxE test statistics at heritable sites of the confounding environment. Relatively smooth non-linearities, such as squared effects, are easily detected by regression modeling before using LEMMA (see Materials and Methods) and can then be included as covariates [indicated by (+SQE) in Figure 2]. This procedure produced well-calibrated test statistics for all methods in simulation (Figure 2C).

In Figure 2B we compare the GxE association test statistics from our analysis of logBMI in the UK Biobank with and without adjusting for detected squared effects. Although we detected squared effects for 30 of the 42 environmental variables (significance level 0.01; Bonferroni correction for multiple testing), the ES obtained from the two analyses was almost identical (Pearson $r^{2}>0.999$). Because the additional variance explained collectively by the squared effects was negligible (incremental $R^{2}<0.00001$), it would be surprising if this was not the case. Negative $\mathrm{lo}g_{10}\left(p\right)$ values from the two analyses were also highly correlated (Pearson $r^{2}=0.961$), although there were small changes in the p values at the *FOXO3* locus (which remained genome-wide significant in both analyses) and at the *SNAP25* locus [which was genome-wide significant in the (−SQE) analysis only]. We therefore conclude that the influence from this form of confounding in our analysis of logBMI was minor. However, because the cost to this procedure is small, LEMMA uses the (+SQE) strategy by default for all analyses of UK Biobank traits.

### GxE Interaction Analysis in the UK Biobank

We applied LEMMA to characterize GxE interactions in BMI (logBMI), SBP, DBP, and PP by using a set of 42 environmental variables similar to those used in previous analyses,^7^^,^^8^^,^^50^ including data on smoking, hours of TV watched, Townsend index, physical exercise, and alcohol consumption (see Materials and Methods and Table S1).

We analyzed GxE heritability due to multiplicative effects with the ES by using both M=639,005 genotyped SNPs and M=10,270,052 common imputed SNPs (MAF ≥0.01 in the full UK Biobank cohort) stratified by MAF and LDscore into 20 components. Using imputed SNPs, we estimated GxE heritability of 9.3%, 12.5%, 3.9%, and 1.6% for logBMI, PP, SBP, and DBP, respectively (see Table 1). On genotyped SNPs, the GxE heritability estimates were slightly lower for logBMI and PP ($h_{\mathrm{GxE}}^{2}=8.6\%$ and $h_{\mathrm{GxE}}^{2}=11.1\%$, respectively) and almost identical for SBP and DBP (see Table S2). For all traits, the heritability of additive SNP effects was slightly higher on imputed data, consistent with previous results.^52^

**Table 1:** Partitioned Heritability Estimates for Four Quantitative Traits in the UK Biobank

**Trait**	$h_{G}^{2}$**(SE)**	$h_{\mathrm{GxE}}^{2}$**(SE)**
logBMI	0.274 (0.056)	0.093 (0.028)
INT (BMI)	0.278 (0.056)	0.059 (0.024)
BMI	0.268 (0.055)	0.137 (0.031)
PP	0.228 (0.051)	0.125 (0.028)
SBP	0.251 (0.05)	0.039 (0.023)
DBP	0.254 (0.05)	0.016 (0.02)

Heritability estimates obtained with common imputed SNPs (MAF >0.01 in the full UK Biobank cohort) with RHE-LDMS. GxE heritability estimates were obtained via the ES from each model fit. All analyses controlled for the same covariates used in the WGR analysis (including the top 20 principal components). Abbreviations are as follows: SE, standard error estimated with the block jackknife (see Materials and Methods); $h_{G}^{2}$, heritability due to additive genetic effects; $h_{\mathrm{GxE}}^{2}$, heritability due to multiplicative GxE effects; RHE, randomized HE regression;^27^^,^^28^ LDMS, SNPs stratified by minor allele frequency and LDscore (20 components); INT, inverse normal transform applied to males and females separately.

When working with quantitative traits, it can be hard to choose an optimal transformation or scale for each trait. Tyrell et al.^26^ analyzed BMI by using the raw scale and then also by transforming to a standard normal distribution. They observed larger interaction effects on the raw scale and suggested that this was due to larger variance in BMI in individuals in the high-risk environment groups, which causes heteroskedasticity and inflates effect estimates. In addition to our main analysis, which used log BMI, we re-ran LEMMA by using the raw BMI measurement and then also by transforming to a standard normal distribution in females and males separately. These results are presented in Table 1 and agree with the results of Tyrell et al.:^26^ estimates of GxE heritability on the raw, log, and inverse normal scales were 13.7%, 9.3%, and 5.9%, respectively.

**Figure 3:**
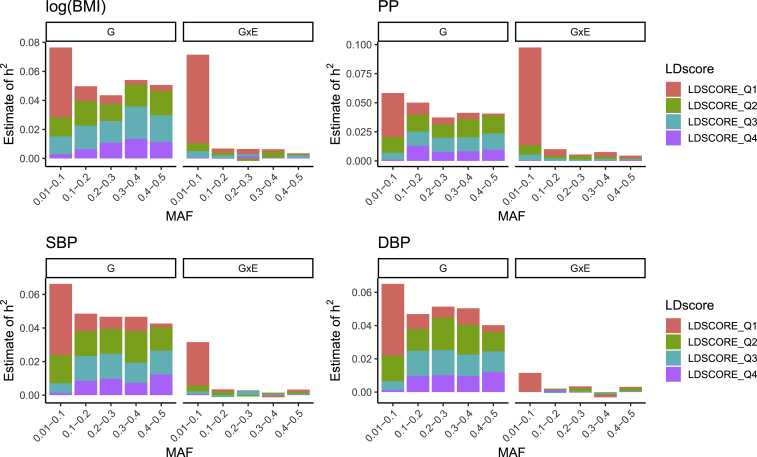
Partitioned Heritability Estimates for Four Quantitative Traits in the UK Biobank Heritability estimates partitioned into additive genetic and multiplicative GxE interaction effects for four quantitative traits in the UK Biobank with approximately 280,000 unrelated white British individuals (see Table S1) and M=10,270,052 common imputed SNPs (MAF >0.01 in the full UK Biobank cohort). Multiplicative GxE interactions were computed with the ES from each model fit. Heritability estimation was performed via a multi-component implementation of RHE regression^27^^,^^28^ with SNPs stratified into 20 components (5 MAF bins and 4 LD score quantiles).

Previous work on models of natural selection has suggested that the variance explained by additive SNP effects should be uniformly distributed as a function of MAF in a neutral evolutionary setting^54^ and that enrichment of the variance explained by low-frequency SNPs is evidence for negative selection. For all four traits, we found that variance explained by the additive genetic effects of low-frequency SNPs (MAF <0.1) was slightly elevated, consistent with previous observations of negative selection^46^ (Figure 3). Additionally, the distribution of additive genetic effects by MAF for logBMI was qualitatively similar to that found by GREML-LDMS in a previous study.^52^ In contrast, we found that variance explained by GxE effects was overwhelmingly attributed to low-frequency SNPs (MAF <0.01), especially those with low LD. However, we are not aware of any evolutionary theory that has been extended to model the MAF distribution of GxE effects.

**Figure 4:**
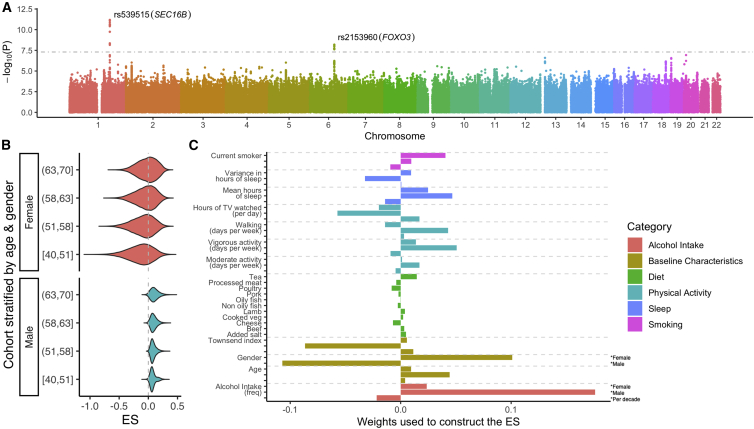
GxE Analysis of logBMI in the UK Biobank (A) LEMMA association statistics testing for multiplicative GxE interactions at each SNP. The horizontal gray line denotes $\left(p=5\times 10^{-8}\right)$, and p values are shown on the $-\mathrm{lo}g_{10}$ scale. (B) Distribution of the environmental score (ES), stratified by gender and age quantile. (C) Weights used to construct the ES. Dietary variables have a single weight shown on the per-standard-deviation (SD) scale. “Gender” has two weights; a gender-specific intercept for women (first) and for men (second). Remaining non-dietary variables have three weights: (1) a per-SD effect for women only, (2) a per-SD effect for men only, and (3) a per-SD, per-decade effect, which is the same for both genders. SD for the male- and female-specific weights is computed for each gender separately. Age is computed as the number of decades aged from 40 years. See Materials and Methods for details.

For logBMI, we estimated an ES that put high weight on alcohol intake frequency, Townsend index, and physical activity measures (Figure 4C). Almost all of the non-dietary environmental exposures had a higher effect in women than in men; smoking status was the one exception. This is reflected in the facts that (A) the ES has much higher variance in women and (B) those with a negative ES were almost all female (97%) (see Figure 4B). When comparing the characteristics of those in the bottom 5% of the ES to the whole cohort (by using the mean for continuous variables and the mode for categorical), we found that those in the bottom 5% were predominantly female (100% versus 53%), younger (51 years versus 56 years), had a higher Townsend deprivation index (0.91 versus −1.74), drank less often (“special occasions” versus “once or twice a week”), and watched more TV (3.28 h versus 2.69 h of TV daily) (Table S3). We note that positive values of the Townsend index indicate material deprivation, whereas negative values indicate relative affluence.

Previous cross-sectional studies have reported GxE interactions between a linear predictor formed from BMI-associated SNPs and alcohol intake frequency,^55^ Townsend index,^26^^,^^55^ physical activity measures,^5^^,^^26^^,^^55^^,^^56^ and time watching TV,^26^^,^^55^^,^^56^ all of which had high relative weight in the logBMI ES. An alternative approach from Robinson et al.^6^ binned samples according to their environmental exposure (e.g., age) and tested for significant differences in SNP heritability by using a likelihood ratio test. They reported strong interaction effects with age in a cohort of 43,407 individuals whose ages spanned 18−80 years but only reported significant interactions with smoking in the UK Biobank interim release. This suggests that we might expect age to play a more dominant role in the logBMI ES in a cohort that included younger individuals. Finally, one category that is notably down weighted is the contribution from dietary variables. Although significant interactions with fried food consumption^57^ and sugar sweetened drinks^58^ have previously been reported in a cohort of US health professionals, these dietary variables were not included in the diet questionnaire used by the UK Biobank.

The ES for PP was dominated by the effects of age and gender (age, ${\text{age}}^{2}$, age-x-gender, and gender together explained 94.9% of variance in the ES). The magnitude of the ES was strongly associated with increased age,^59^ whereas the sign of the ES was strongly associated with gender, implying that GxE effects were stronger in the elderly but acted in the opposite direction in men and women (Figure S7).

Similarly, we observed that variance of the ES increased with age in both SBP and DBP, but instead of age itself being highly weighted, we found that age interactions with other environmental variables were most important for explaining variation in the ES. Specifically, for SBP, we found that age interactions with smoking, Townsend index, and alcohol frequency explained 86% of variance in the ES (Figure S8). When compared to the cohort average, we found that participants in the top 5% of the SBP ES were older (63 years versus 58 years), had a higher Townsend deprivation index (1.2 versus −1.74), and were more likely to smoke (59% versus 9%), whereas those in the bottom 5% were also older (65 years versus 58 years), predominantly female (91.5%), rarely drank alcohol (43.9% drank “never”), and a had low Townsend deprivation index (−2.9 versus −1.74) (Table S4).

Finally, we observed notably higher variance in the ES for DBP among men, most of which appeared to be driven by high gender-specific weights for smoking status and alcohol frequency (Figure S9). We further observed that alcohol frequency and smoking status became increasingly influential with age. The total SNP-GxE heritability for this ES, however, was quite low.

When testing for significant GxE interactions between the estimated ESs and imputed markers across the genome, we observed that use of the robust standard errors made a noticeable difference to the calibration of LEMMA (Figure S10 and Table S5). We identified two loci for logBMI (Figure 4), one locus for DBP (Figure S9A), and zero loci for SBP and PP by using a threshold of $5\times 10^{-8}$ for genome-wide significance (Table 2). This table also includes results from a standard linear regression GWAS test at the three loci. Table S6 provides full parameter estimates of the environmental, SNPs, and SNP-ES effects.

**Table 2:** Loci with Genome-wide Significant GxE Interaction Effects with the ES

**SNP**	**rs539515**	**rs2153960**	**rs8090962**
Trait	log(BMI)	log(BMI)	DBP
Chr	1	6	18
BP	177889025	108988184	56694404
A0	A	G	A
A1	C	A	G
AF	0.21	0.71	0.44
Nearest gene	*SEC16B*	*FOXO3*	*OACYLP / SEC11C*

**Standard GWAS Tests**			

$\beta_{G}$ (SE)	0.0043 (0.0003)	0.0014 (0.0003)	−0.00001 (0.0192)
$p\;\mathrm{valu}e_{G}$	$5.7\times 10^{-51}$	$1.1\times 10^{-6}$	$1.0\times 10^{+0}$

**LEMMA Association Tests**			

$\beta_{G}$ (SE)	0.0254 (0.0016)	0.0087 (0.0015)	0.0011 (0.0015)
$\beta_{\mathrm{GxE}}$ (SE)	−0.0117 (0.0017)	−0.0098 (0.0017)	0.0087 (0.0016)
$p\;\mathrm{valu}e_{G}$	$1.6\times 10^{-60}$	$1.6\times 10^{-8}$	$4.5\times 10^{-1}$
$p\;\mathrm{valu}e_{\mathrm{GxE}}$	$6.5\times 10^{-12}$	$6.5\times 10^{-9}$	$3.6\times 10^{-8}$

Independent loci with genome-wide significant ($p<5\times 10^{-8}$) GxE interaction effects with the environmental score (ES). Loci at least 0.5 cM apart were judged to be independent. SNP effect sizes reported on a per-SD scale. SNP locations follow the GrCh37 human genome assembly. All loci had an IMPUTE info score >0.99. Abbreviations are as follows: BP, base pairs; A0, reference allele; A1, alternative allele; AF, reference allele frequency; SD, standard deviation.

For logBMI, LEMMA identified GxE interactions at rs2153960 ($p=6.5\times 10^{-9}$; Figure S11) and at rs539515 ($p=6.5\times 10^{-12}$; Figure S12). The SNP rs2153960 is an intron in *FOXO3* and has been previously associated with insulin-like growth factor 1 (IGF-1) concentration in a cohort of 10,000 middle-aged Europeans.^60^ IGF-1 is known to be a central mediator of metabolic, endocrine, and anabolic effects of growth hormones and is also involved in carbohydrate homeostasis.^60^ The patterns of main effect association and GxE association show considerable overlap (Figure S11A). This SNP did not reach genome-wide levels of significance with the standard linear regression GWAS test (Table 2).

The SNP rs539515 is located 6 kb downstream of *SEC16B*. The patterns of main effect association and GxE association are very similar (Figure S12A). Multiplicative GxE interactions have been reported at *SEC16B* with multiple environmental variables in a similar analysis in the UK Biobank^7^ and with physical activity separately in Europeans^5^ (p=0.025) and in Hispanics^61^ $\left(p=8.1\times 10^{-5}\right)$. Highly significant variance effects $\left(p=3.88\times 10^{-17}\right)$, which can be indicative of GxE, have also been reported at the *SEC16B* locus via N=456,422 Europeans in the UK Biobank.^62^ *SEC16B* transcribes one of the two mammalian orthologs of SEC16, which has a key role in organizing endoplasmic reticulum exit sites by interacting with COPII components.^63^ Although several GWASs have identified associations between *SEC16B*,^64^^,^^65^ the relevance of *SEC16B* to BMI is not well characterized.^66^ Some evidence exists to suggest that *SEC16B* has role in the transport of peroxisome biogenesis factors; peroxisomes are an organelle involved in the catabolism of long-chain fatty acids found ubiquitously in eukaryotic cells. Previous authors^65^ have also speculated that *SEC16B* might play a role in the transport of appetite regulatory peptides; however, we are not aware of any evidence for this theory.

The DBP-associated SNP is rs8090962, but it only just passes our threshold for significance and we are least confident that this is a true GxE association for a few reasons. The SNP is located within an enhancer, approximately 100 kb downstream of *SEC11C* and 50 kb upstream of *ZNF532*. Neither gene has previously been associated with blood pressure traits. There is some evidence of a main effect close by (Figure S13A), but the pattern of main effect association does not coincide well with the pattern of GxE association. In addition, the pattern of GxE association by genotype (Figure S13B) shows a striking cross over by genotype between extremes of the ES. We have observed above that our test statistics are very slightly inflated, so this could be a false positive association.

### Relationship of Genetic PCs and Environmental Scores

We regressed the estimated ESs against the PCs for each of the four UK Biobank traits, and the results are included in Table S7. We found some significant associations, mostly with PC5, which seems to correlate with North-South geography in the UK.^67^ To explore further, we also re-ran the heritability analysis by including interaction terms of the ES with the genetic PCs as control variables, but the results were almost unchanged (see Table S8).

### Comparison of the LEMMA ES with a Marginal ES

For each trait, we used least-squares regression to compute a linear model fit using all of the non-genetic covariates used in the LEMMA analysis. We then constructed an ES (referred to as ${\text{ES}}_{\mathrm{marginal}}$) by using the marginal environmental effects from this model fit. The correlation between the LEMMA ES and ${\text{ES}}_{\mathrm{marginal}}$ was −0.062,−0.019,−0.297, and −0.088 for logBMI, PP, SBP, and DBP, respectively, suggesting that these vectors are quite dissimilar. Figure S14 shows a comparison of the interaction weights used to construct the LEMMA ES and ${\text{ES}}_{\mathrm{marginal}}$ for each of the four traits. Visually, the weights learnt through each approach look quite distinct. In particular, age, ${\text{age}}^{2}$, and age×gender have much higher relative weights in ${\text{ES}}_{\mathrm{marginal}}$ than in the LEMMA ES.

### Comparison of Methods on UK Biobank Data

To compare LEMMA with existing single SNP methods, we also ran StructLMM, the F-test, and the robust F-test on logBMI by using the same set of environmental variables as used by LEMMA (but not including the significant squared environments as covariates). Manhattan plots are displayed in Figure S15. Test statistics from both the F-test ($\lambda_{\mathrm{GC}}=1.37$) and StructLMM ($\lambda_{\mathrm{GC}}=1.235$) were substantially inflated when compared to the robust F-test and LEMMA ($\lambda_{\mathrm{GC}}=1.03$ and $\lambda_{\mathrm{GC}}=1.062$, respectively; see Table S5), suggesting that StructLMM does not properly control for heteroskedasticity. There are clear differences between the four methods, especially among SNPs with suggestive evidence of GxE interaction results (Figure S16). LEMMA did not find the FTO locus, StructLMM and F-test did not find the SEC16B locus, and the robust F-test only found the FTO locus.

LEMMA relies on the assumption that all GxE interaction effects for a single trait share a common ES, and we have shown in simulation that, when this assumption holds, LEMMA achieves substantial increases in power. However, we would expect LEMMA to have little power to detect SNPs that interact with a combination of environments that are not well correlated with the genome-wide ES estimated by LEMMA. *FTO* seems to be one clear example of this. We extracted an estimate of the SNP-specific interaction profile at *FTO* by using the robust F-test (Materials and Methods), and we found that its correlation with LEMMA’s ES was low (Pearson $r^{2}=0.3$). In comparison, a similar analysis at *SEC16B* and *FOXO3* yielded much higher correlations (Pearson $r^{2}=0.725$ and $r^{2}=0.713$, respectively).

## Discussion

In this study, we proposed a WGR method, LEMMA, that estimates a single ES that interacts with SNPs across the genome. In simulation, we have demonstrated that the ES can be used to compute well-calibrated p values of the multiplicative interaction effect at each SNP. LEMMA is also able to quantify the trait variance attributable to MAF- and LD-stratified interaction effects of the ES.

In our analyses of four quantitative traits in the UK Biobank, we have demonstrated that GxE effects among common imputed SNPs make a non-trivial contribution to the heritability of logBMI and PP (9.3% and 12.5%, respectively). Our stratified heritability analysis has suggested that GxE interactions for these traits are mostly driven by low-frequency variants. Our analysis identified three loci with statistically significant GxE interaction effects. As far as we are aware, two of these loci, rs539515 (*FOXO3*) and rs8090962, are novel, and for the other, rs539515 (*SEC16B*), we show stronger evidence for statistically significant GxE interaction effects than the previous study.^7^

Robinson et al.^6^ have previously attempted to quantify the contribution of GxE interactions to the heritability of BMI in a study performed on imputed SNPs from the interim UK Biobank release. Using the GCI-GREML model implemented in GCTA^68^ and eight environmental variables that included measures of smoking, hours of TV watched, and alcohol frequency, Robinson et al.^6^ reported that only smoking had significant GxE heritability (4.0%). In contrast, the ES estimated for logBMI in our analysis had non-zero contributions from many environmental variables, including hours of TV watched and smoking, suggesting that multiple environmental variables can influence the genetic predisposition to BMI. Modeling these environmental variables jointly allowed LEMMA to capture a combination whose GxE interactions explained 9.3% of heritability.

We have also evaluated the performance of three existing single SNP methods (StructLMM, the F-test, and a robust F-test) both in simulation and on logBMI from this same dataset. In simulation with large datasets, we observed that StructLMM and the F-test had similar performance, an observation that also held in our analysis of logBMI. Both of these methods appeared vulnerable to heteroskedasticity, which we showed is likely to occur in traits with non-trivial GxE heritability. A simple adjustment, using “robust” or Huber-White variance estimators, solved this problem. The two F-test methods further benefit from a wealth of existing theory^41^ and, being theoretically simpler than StructLMM, could be easily implemented as an R-plugin with PLINK^69^ (for example^37^). In our opinion, the robust F-test is therefore the most appropriate of the three single SNP methods to model GxE effects with tens of environments in biobank-scale datasets.

Although LEMMA represents a method with increased power to detect GxE interaction effects, our approach does have some caveats. First, the gain in power is dependent on a strong assumption on the underlying genetic architecture. Although our analysis suggests that this does hold to some extent for PP and logBMI, this may not be the case for other traits.

In addition, LEMMA only estimates the proportion of phenotypic variance that is explained by interactions with this ES and we do not claim that this captures all the GxE heritability of a trait. If relevant GxE environments are not included in the analysis, and these environments have low correlation to the environments that are included, then LEMMA cannot account for them and will most likely underestimate the true GxE heritability. Unobserved environments can cause trait variance to depend on genotype^8^ (see Figure S19), and extending LEMMA in this direction is left for future work.

LEMMA has the requirement that none of the environmental variables have any missing values. This could lead to a reduction in samples size if many environmental variables are included. If the amount of missing data is small, it should not pose a big problem, and missing data imputation methods are also an option. If LEMMA is applied in situations where the missing data structure is related to the phenotype of interest, then this could cause bias in the results.

Despite much effort to provide an efficient implementation, the LEMMA algorithm is still computationally demanding. Using randomized HE regression to estimate an improved initialization of the interaction’s weights may help to reduce runtime and is an avenue that we are currently pursuing.

Finally, for simplicity, LEMMA currently searches only for GxE interactions with a single linear combination of environments. Generalizing the LEMMA approach to several orthogonal linear combinations or using functional annotation to restrict the SNPs that each ES interacts with may yet yield more power to identify interactions in complex traits and explain more phenotypic variation.

## Declaration of Interests

J.M. owns stocks and stock options in Regeneron Pharmaceuticals.

## Web Resources

StructLMM as implemented in LIMIX 2.0.0, https://github.com/limix/limix

## Data and Code Availability

The genetic and phenotype datasets generated by UK Biobank analyzed during the current study are available via the UK Biobank data access process. The resource is available to all bona fide researchers from academic, charity, public, and commercial sectors for all types of health-related research that is in the public interest: there is no preferential or exclusive access for any person. More details are available at http://www.ukbiobank.ac.uk/register-apply/. Pre-compiled binaries and source code for the LEMMA method are available from https://jmarchini.org/lemma/.

## Supplemental Data

Document S1. Figures S1–S19, Tables S1, S2, S5, and S7–S9, and Supplemental Notes

Table S3 Summary Statistics of the Environment for Participants in the Top and Bottom 5% of the Environmental Score for log(BMI)

Table S4 Summary Statistics of the Environment for Participants in the Top and Bottom 5% of the Environmental Score for SBP

Table S6 Effect Sizes, Standard Errors, and p Values of All Covariates Used in a “Vanilla GWAS” Analysis of the Three SNP Traits with Genome-wide Significance in the LEMMA of the UK Biobank

Document S2. Article plus Supplemental Information
