# Supplementary material for: Inferring Gene-by-Environment Interactions with a Bayesian Whole-Genome Regression Model
Source: Am J Hum Genet. 2020 Sep 3;107(4):698–713. doi: 10.1016/j.ajhg.2020.08.009 (PMC7536582; doi:10.1016/j.ajhg.2020.08.009)
Supplement: Document S1. Figures S1–S19, Tables S1, S2, S5, and S7–S9, and Supplemental Notes [file mmc1.pdf]

The American Journal of Human Genetics, Volume *107*

Supplemental Data

Inferring Gene-by-Environment Interactions with a Bayesian Whole-Genome Regression Model

Matthew Kerin and Jonathan Marchini

Supplementary Figures

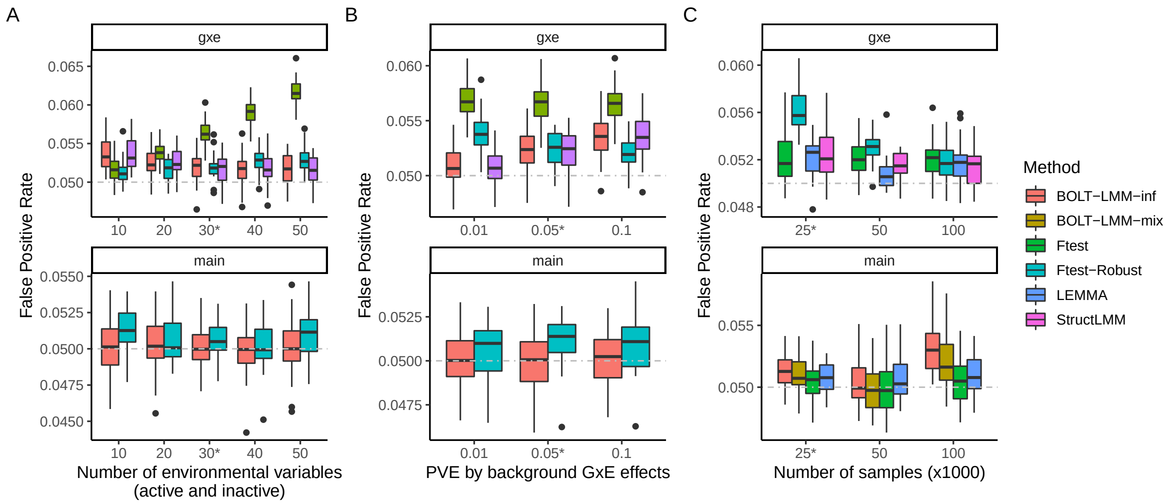

Figure S1: **False positive rates on simulated datasets**. False positive rate (FPR) for SNP main effects tests (bottom) and SNP GxE interaction tests (top) at null SNPs in the second half of each chromosome, whilst varying (a) the number of environmental variables, (b) proportion of trait variance explained by background GxE effects and (c) sample size. The grey line denotes expected FPR. Simulations used genotypes sub-sampled from the UK Biobank and by default contained $N = 25K$ samples, $M = 100K$ SNPs, 6 environmental variables that contributed to the ES and 24 that did not (default parameters denoted by stars). We performed 20 repeats for each scenario. See **Online methods** for full details of phenotype construction.

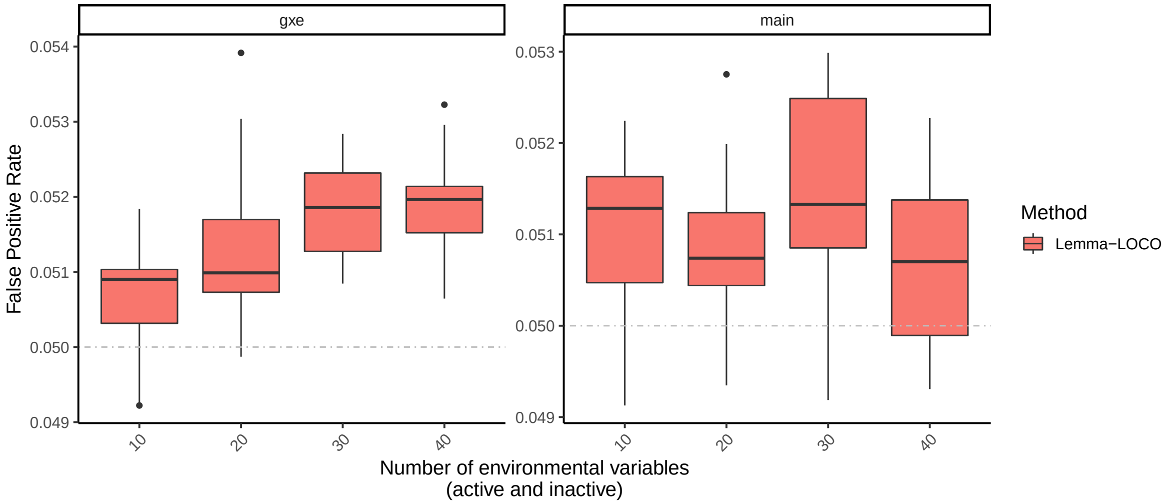

Figure S2: **LEMMA false positive rate in large simulations**. False positive rate (FPR) for SNP main effects tests (right) and SNP GxE interaction tests (left) at null SNPs in the second half of each chromosome, whilst varying the number of environmental variables. The simulation was conducted with $N = 200K$ samples and $M = 400K$ SNPs. The simulated trait was constructed with $10,000$ causal SNPs main effects that explained 20% of variance, and zero causal SNP GxE effects. We performed 20 repeats in each scenario. See **Online methods** for full details of phenotype construction.

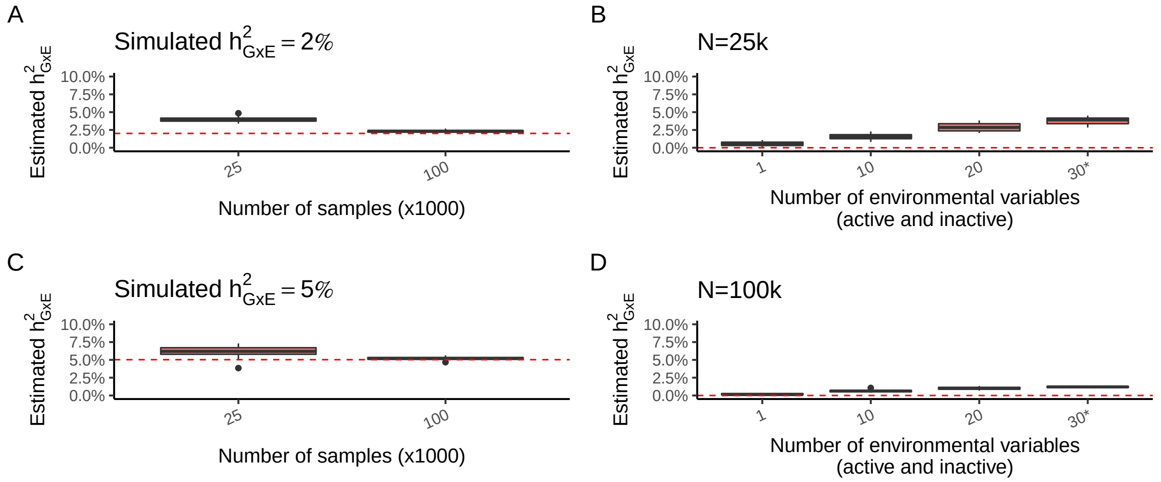

Figure S3: **Estimation of GxE heritability.** Estimates of SNP-GxE heritability whilst varying the number of environmental variables (b, d) and sample size (a, c). The red dotted line denotes the true SNP-GxE heritability used whilst constructing the simulation. We observed some upwards bias as the number of environmental variables increases (b, d), which is ameliorated with increased sample size (d). Phenotypes were constructed using $M = 100,000$ SNPs with $M_{\text{causal, main-effects}} = 80,000$ causal main effects and $M_{\text{causal, GxE-effects}} = 40,000$ causal interaction effects. See **Online methods** for full details of phenotype construction.

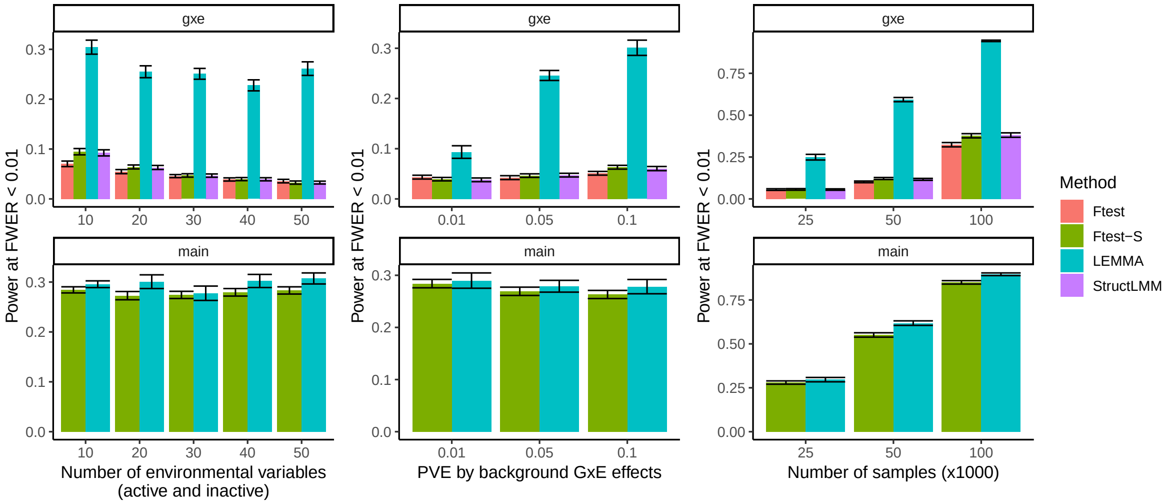

Figure S4: **Power to detect causal SNPs in simulation.** Power to detect SNP GxE interaction effects (top) and SNP main effects (bottom), whilst varying (a) the number of environmental variables, (b) proportion of trait variance explained by background GxE effects and (c) sample size. Power was assessed as the proportion of 60 causal SNPs detected at $p < 0.01$ (Family Wise Error Rate; FWER < 0.01), where causal SNPs main and GxE interaction effects each explained 0.00016% of trait variance. Simulations used genotypes sub-sampled from the UK Biobank and by default contained $N = 25K$ samples, $M = 100K$ SNPs, 6 environmental variables that contributed to the ES and 24 that did not (default parameters denoted by stars). We performed 20 repeats for each scenario. See **Online methods** for full details of phenotype construction.

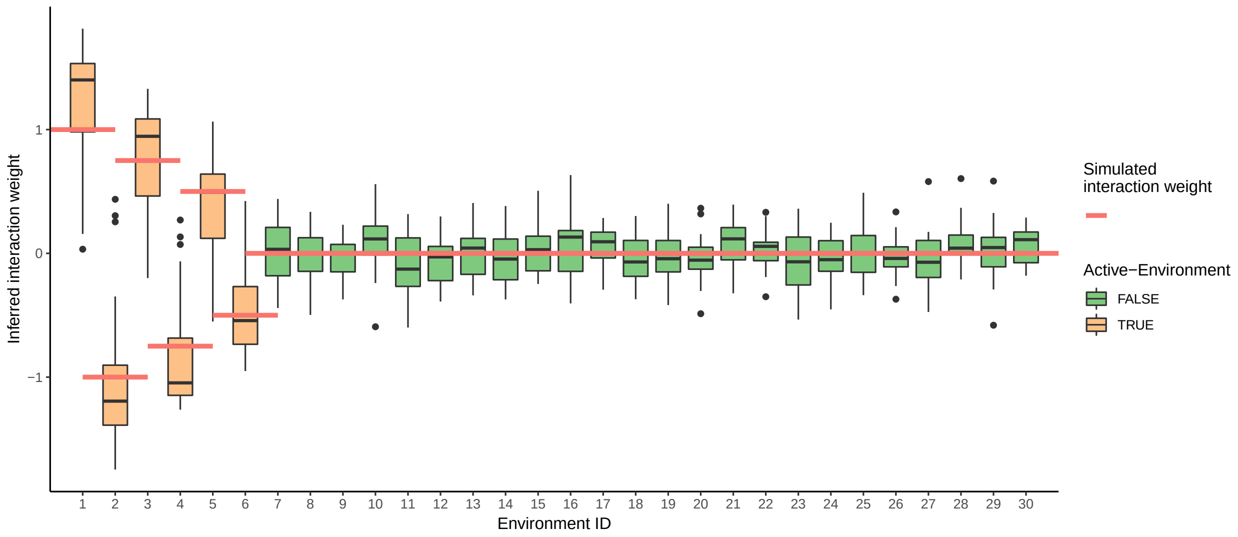

Figure S5: **Estimation of ES weights in simulation.** Boxplots of the environmental score (ES) weights estimated by LEMMA (left) over 20 simulations. Red lines denote true weights used to construct the simulated ES. Simulations performed with $N = 25k$ samples, $M = 100k$ SNPs and $L = 30$ environments (of which 6 were active). Phenotypes were constructed with $M_{\text{causal, main-effects}} = 5000$ SNPs explaining 20% of trait variance and $M_{\text{causal, GxE-effects}} = 2500$ SNPs explaining 5% of trait variance. LEMMA is invariant to a sign change in both the interaction weights and interaction SNP effects, so ES weights are automatically re-scaled such that the largest weight is positive before plotting.

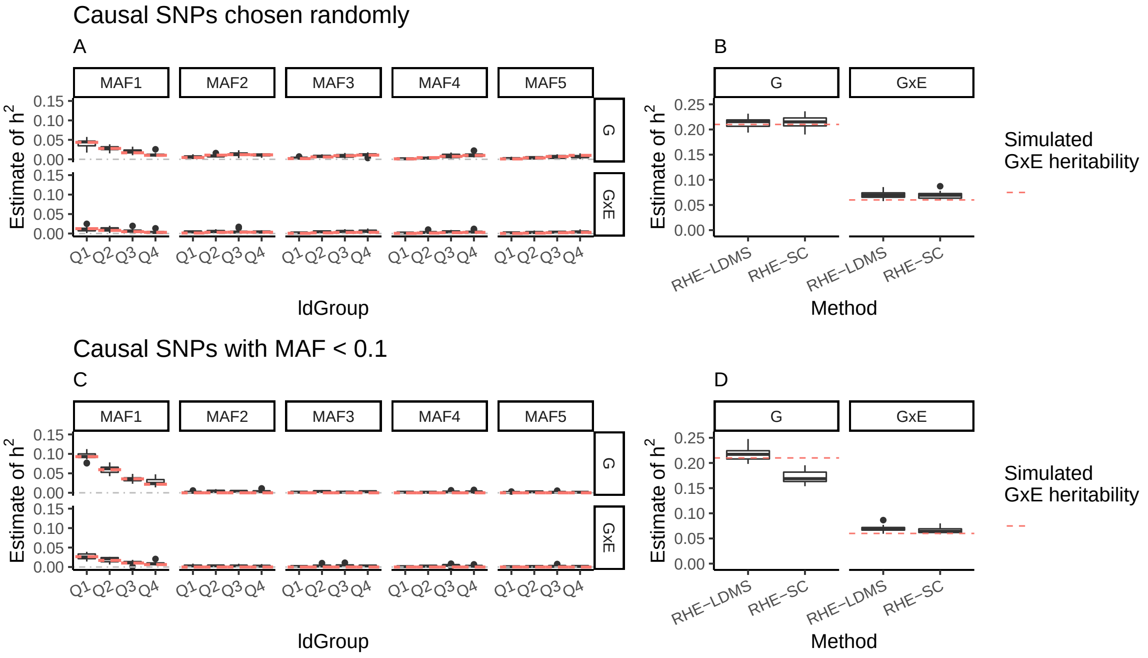

Figure S6: **Heritability estimates stratified by LD and MAF in simulation**. Comparison of heritability estimates using RHE-SC and RHE-LDMS when causal SNPs were drawn (b) at random or (d) only from low frequency (MAF < 0.1) SNPs. Heritability estimates (using RHE-LDMS) stratified by MAF when causal SNPs were drawn (a) at random or (c) only from low frequency (MAF < 0.1) SNPs. Simulations performed with $N = 25K$ samples, $M = 100K$ SNPs and the default simulation parameters described in **Online Methods**. Abbreviations; MAF, minor allele frequency; RHE-SC, randomized HE-regression with a single SNP component[18]; RHE-LDMS, multi-component randomized HE-regression[19].

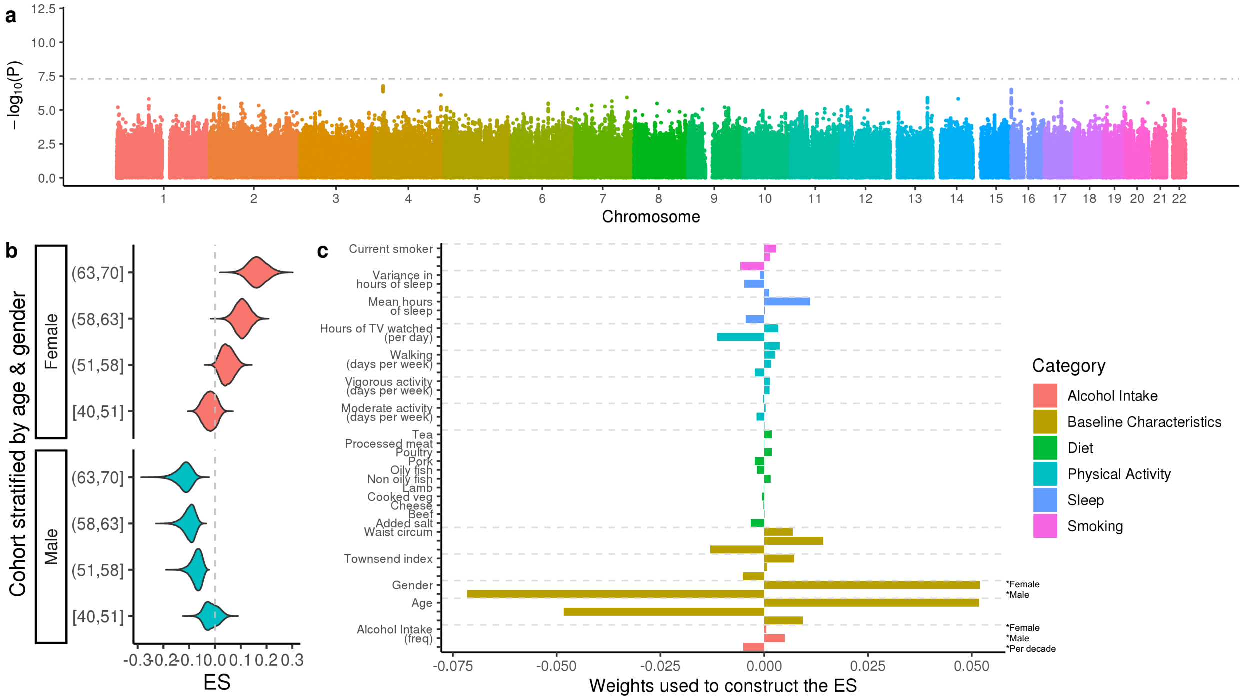

Figure S7: **GxE analysis of PP in the UK Biobank.** (a) LEMMA association statistics testing for multiplicative GxE interactions at each SNP. The horizontal grey line denotes $(p = 5 \times 10^{-8})$, p-values are shown on the $-\log_{10}$ scale. (b) Distribution of the environmental score (ES), stratified by gender and age quantile. (c) Weights used to construct the ES. Dietary variables have a single weight shown on the per standard deviation (s.d) scale. 'Gender' has two weights; a gender specific intercept for women (first) and men (second). Remaining non-dietary variables have three weights; (first) a per s.d effect for women only, (second) a per s.d effect for men only, (third) a per s.d per decade effect which is the same for both genders. s.d for the male and female specific weights is computed for each gender separately. Age is computed as the number of decades aged from 40. See **Online Methods** for details.

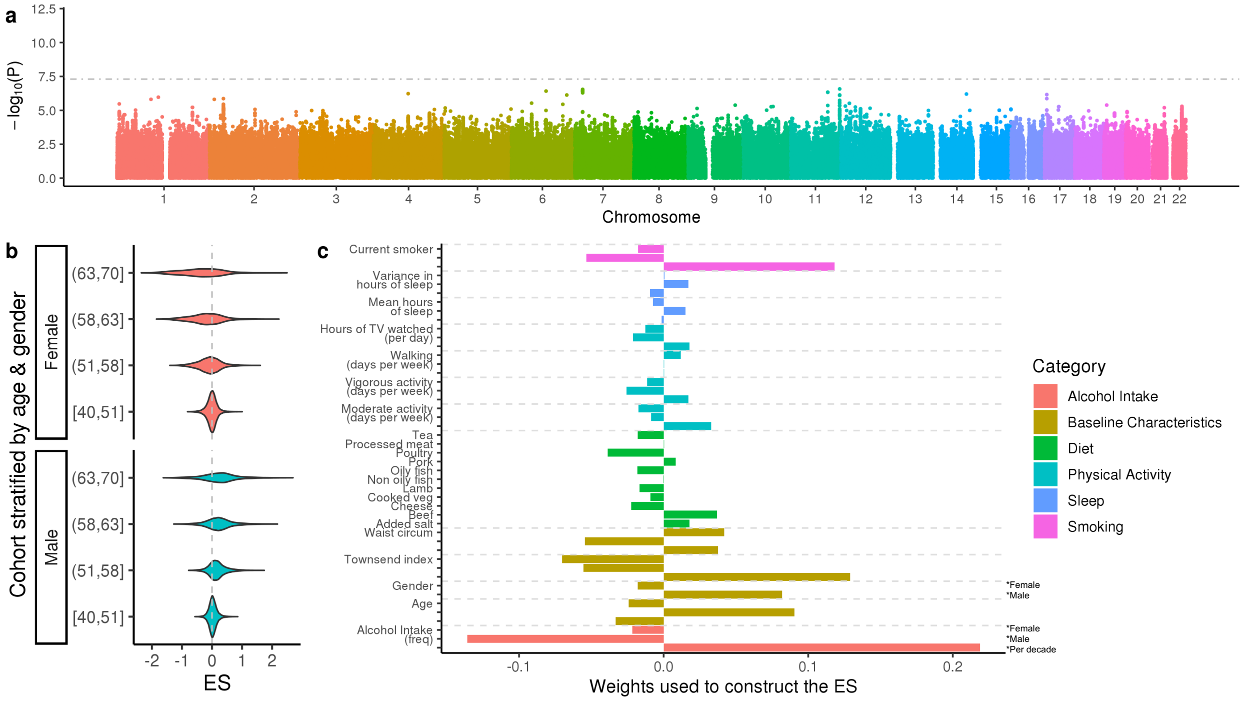

Figure S8: **GxE analysis of SBP in the UK Biobank.** (a) LEMMA association statistics testing for multiplicative GxE interactions at each SNP. The horizontal grey line denotes ($p = 5 \times 10^{-8}$), p-values are shown on the $-\log_{10}$ scale. (b) Distribution of the environmental score (ES), stratified by gender and age quantile. (c) Weights used to construct the ES. Dietary variables have a single weight shown on the per standard deviation (s.d) scale. 'Gender' has two weights; a gender specific intercept for women (first) and men (second). Remaining non-dietary variables have three weights; (first) a per s.d effect for women only, (second) a per s.d effect for men only, (third) a per s.d per decade effect which is the same for both genders. s.d for the male and female specific weights is computed for each gender separately. Age is computed as the number of decades aged from 40. See **Online Methods** for details.

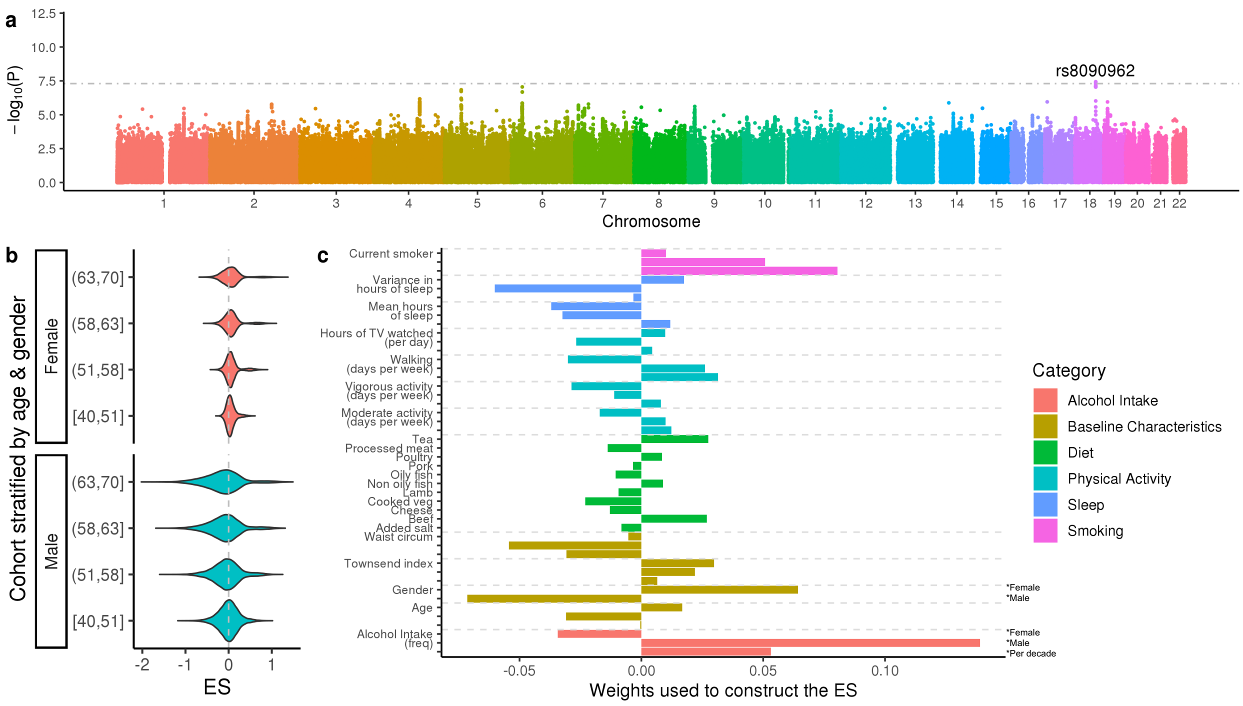

Figure S9: **GxE analysis of DBP in the UK Biobank.** (a) LEMMA association statistics testing for multiplicative GxE interactions at each SNP. The horizontal grey line denotes $(p = 5 \times 10^{-8})$, p-values are shown on the $-\log_{10}$ scale. (b) Distribution of the environmental score (ES), stratified by gender and age quantile. (c) Weights used to construct the ES. Dietary variables have a single weight shown on the per standard deviation (s.d) scale. 'Gender' has two weights; a gender specific intercept for women (first) and men (second). Remaining non-dietary variables have three weights; (first) a per s.d effect for women only, (second) a per s.d effect for men only, (third) a per s.d per decade effect which is the same for both genders. s.d for the male and female specific weights is computed for each gender separately. Age is computed as the number of decades aged from 40. See **Online Methods** for details.

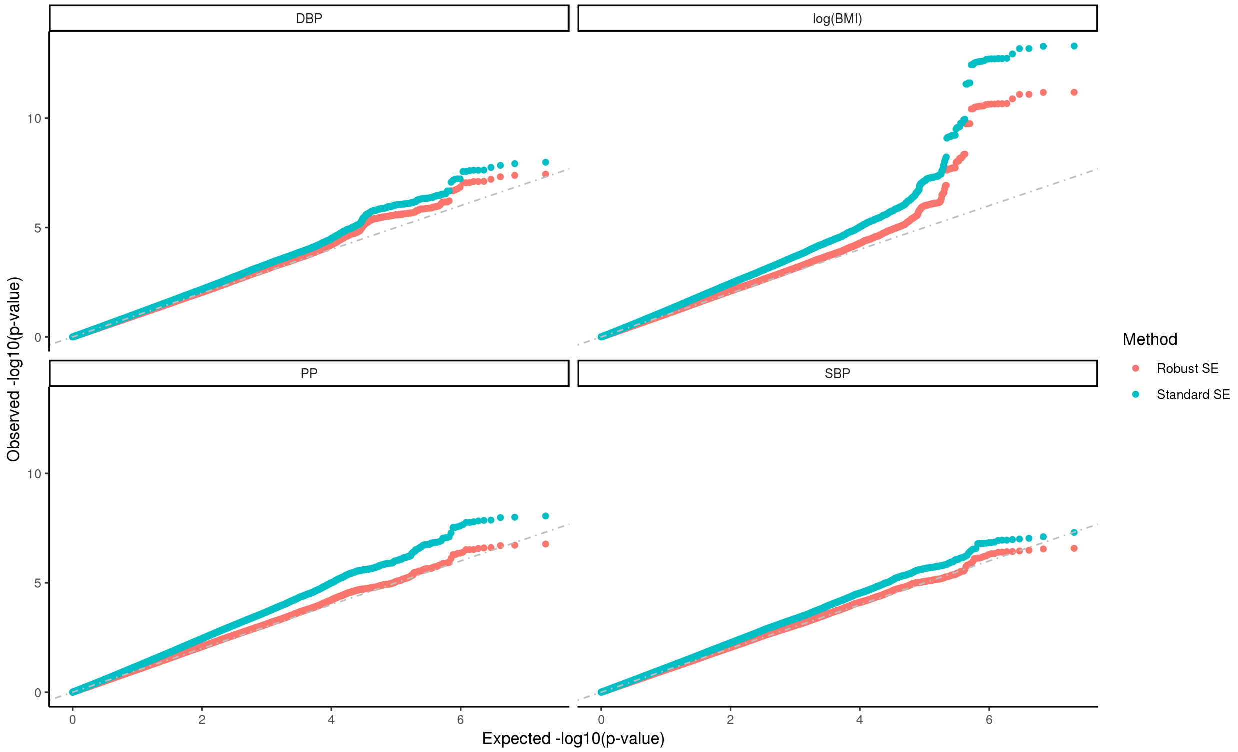

Figure S10: **Effect of using robust standard errors for GxE interaction tests in the UK Biobank.** QQ plots of the observed LEMMA $-\log_{10}(p)$ values for GxE interactions at imputed SNPs for four UK Biobank traits, with and without robust standard errors. The grey dotted line denotes expected $-\log_{10}(p)$-values under a null model. Association tests using 'Robust' standard errors are well calibrated in both homoskedastic and heteroskedastic regimes (see **Online Methods**) and are used in all follow up analysis. Genomic control statistics were $1.275, 1.271, 1.163, 1.111$ for $\log$BMI, PP, SBP and DBP respectively using homoskedastic standard errors and $1.062, 1.047, 1.037, 1.027$ for $\log$BMI, PP, SBP and DBP respectively using robust standard errors.

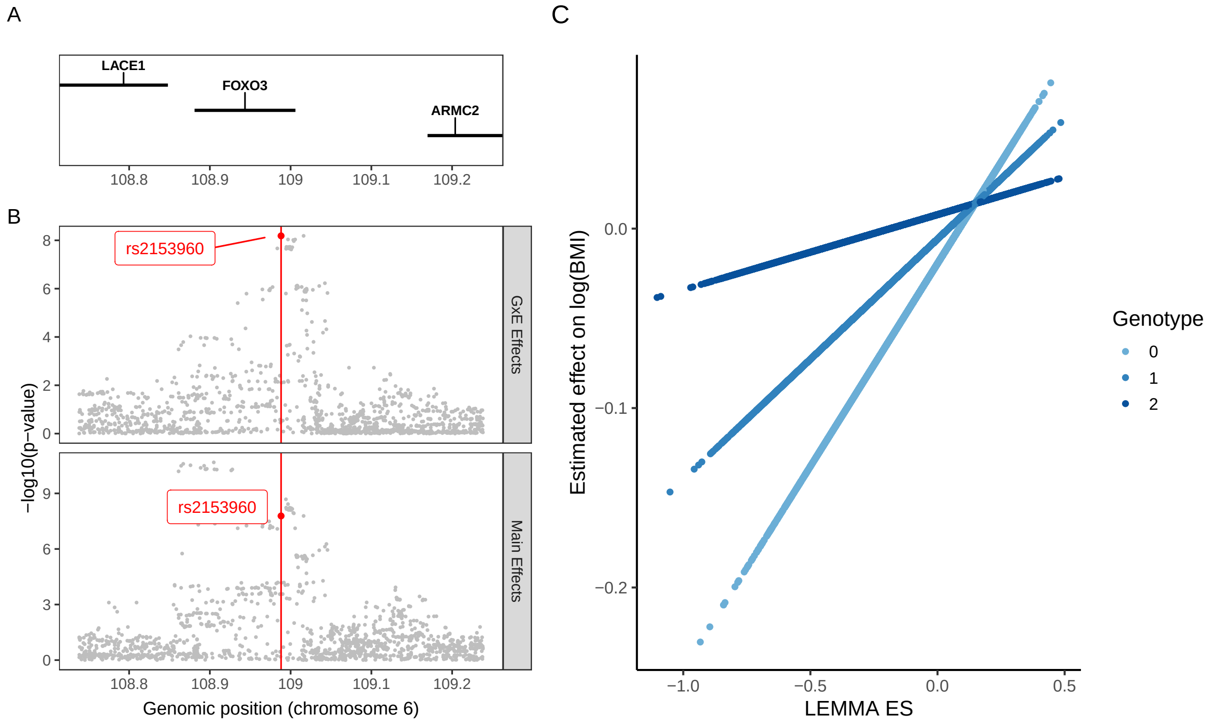

Figure S11: **Estimated GxE effect rs2153960 on $\log$BMI.** (a) Genomic plot of the region surrounding rs2153960, (b) negative $\log_1 0\ p$ values of the main and interaction effects of SNPs within 250KB of rs2153960, (c) the estimated effect of rs2153960 on $\log$BMI as a function of the environmental score (ES).

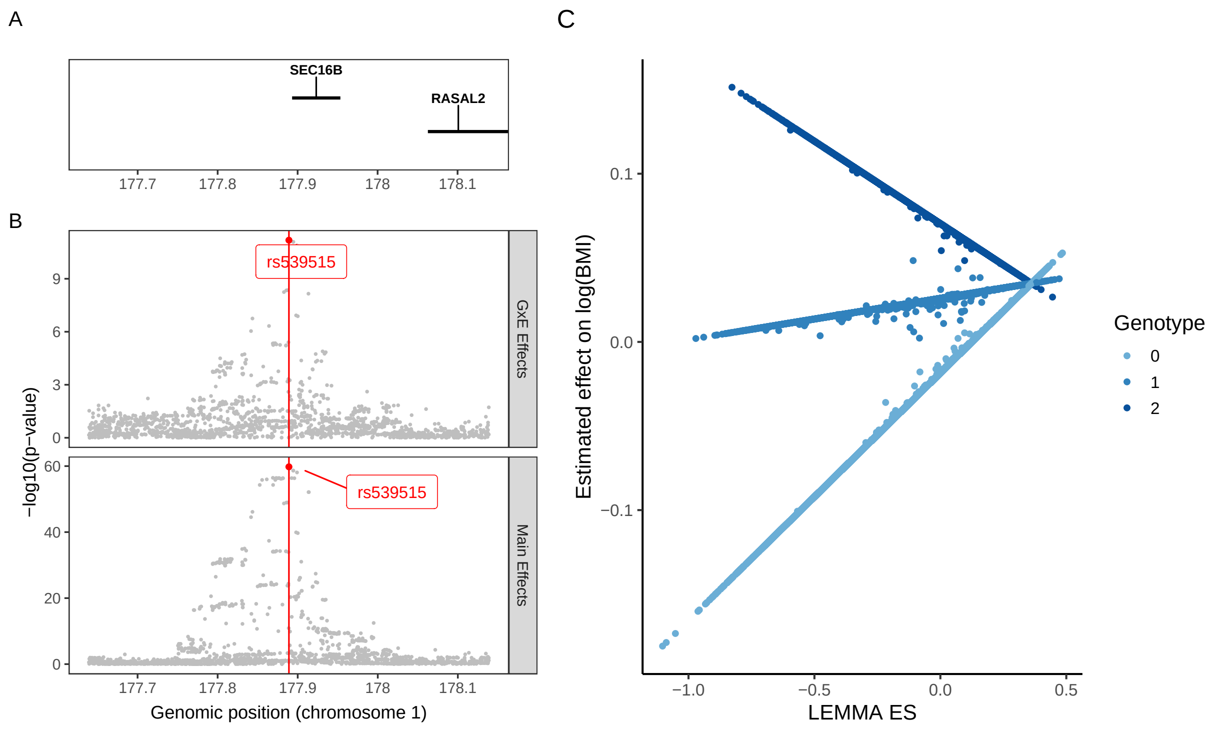

Figure S12: **Estimated GxE effect rs539515 on** $\log$**BMI.** (a) Genomic plot of the region surrounding rs539515, (b) negative $\log_1 0\ p$ values of the main and interaction effects of SNPs within 250KB of rs539515, (c) the estimated effect of rs539515 on $\log$BMI as a function of the environmental score (ES).

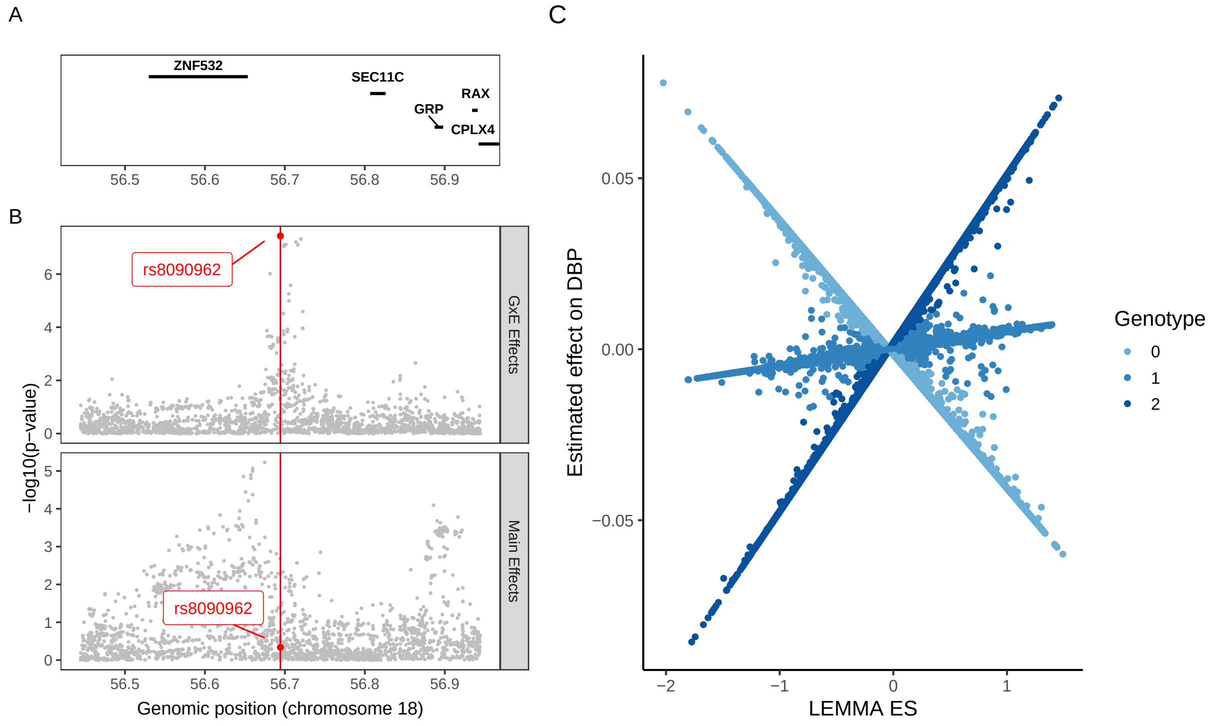

Figure S13: **Estimated GxE effect rs8090962 on DBP.** (a) (a) Genomic plot of the region surrounding rs8090962, (b) negative $\log_1 0\ p$ values of the main and interaction effects of SNPs within 250KB of rs8090962, (c) the estimated effect of rs8090962 on DBP as a function of the environmental score (ES).

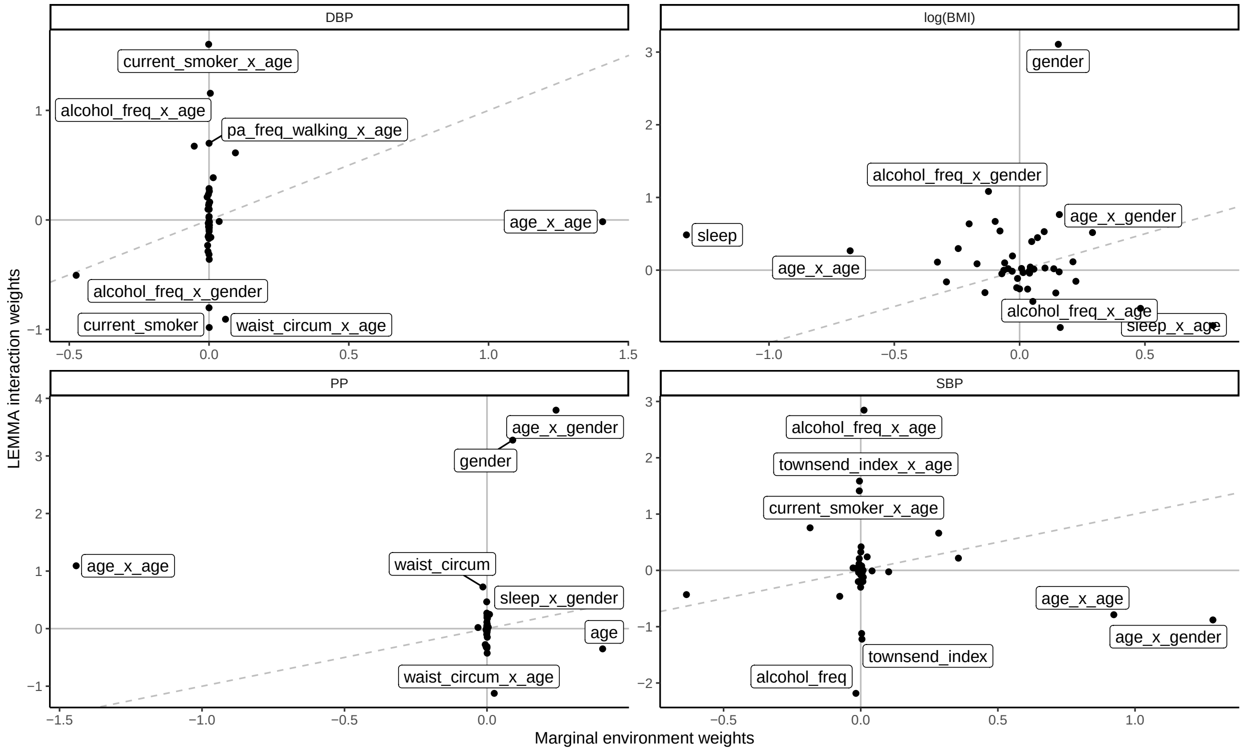

Figure S14: **Comparison of the LEMMA vs marginal environmental score**. Interaction weights of the marginal environmental score were estimated from multivariate linear regression, using all the non-genetic covariates used by LEMMA. Interactions weights were all rescaled so that the corresponding ES had variance one. The dashed grey line represents the $y = x$ line.

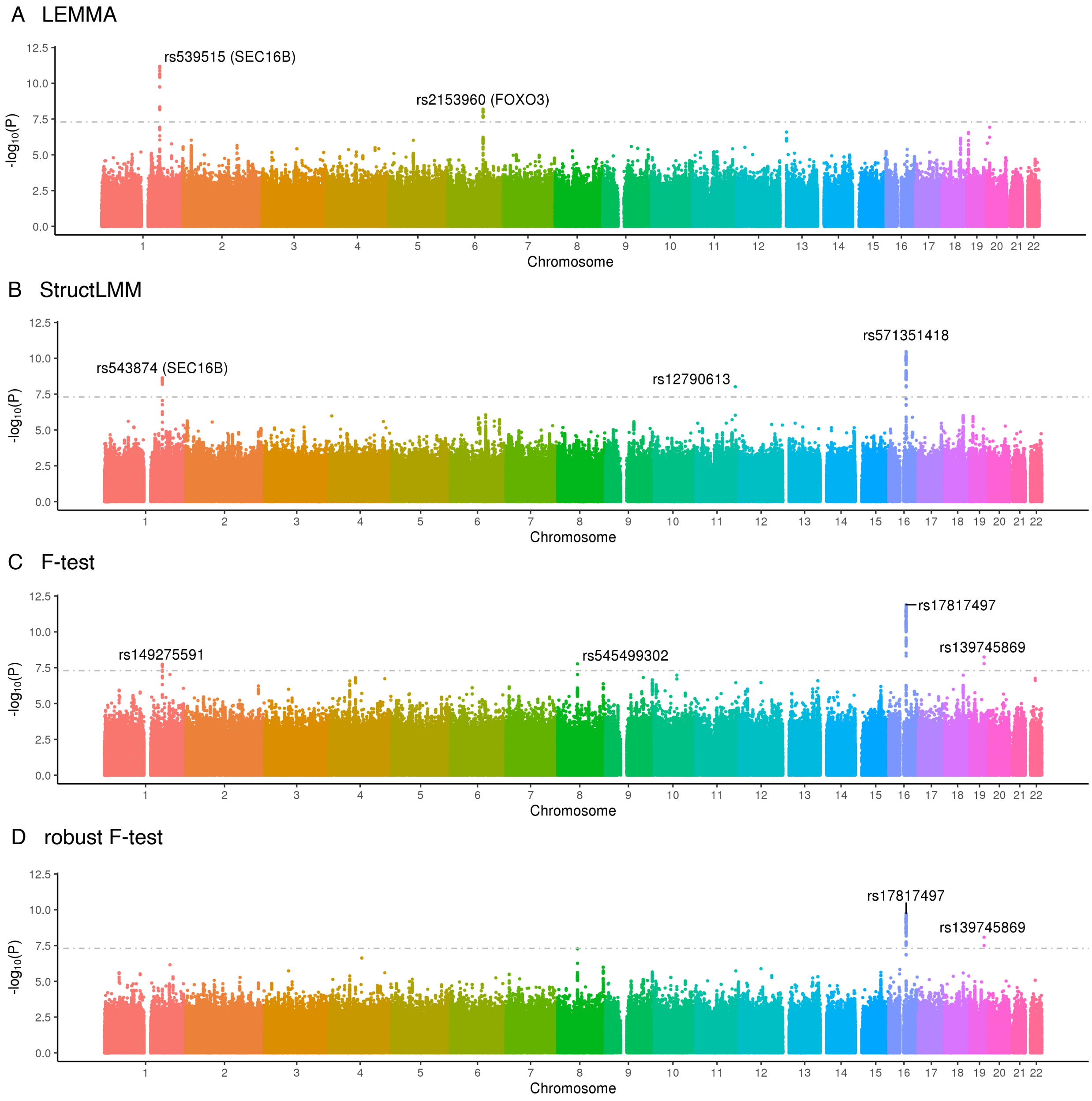

Figure S15: **GxE association statistics for** $\log$**BMI.** Manhattan plots displaying the negative $\log_10$ p values from GxE interaction tests at $10,295,038$ imputed SNPs applied to $\log$BMI in the UK Biobank. GxE interaction tests were computed using (a) LEMMA, (b) StructLMM, (c) the F-test and (d) the robust F-test. The horizontal grey line denotes $(p = 5 \times 10^{-8})$.

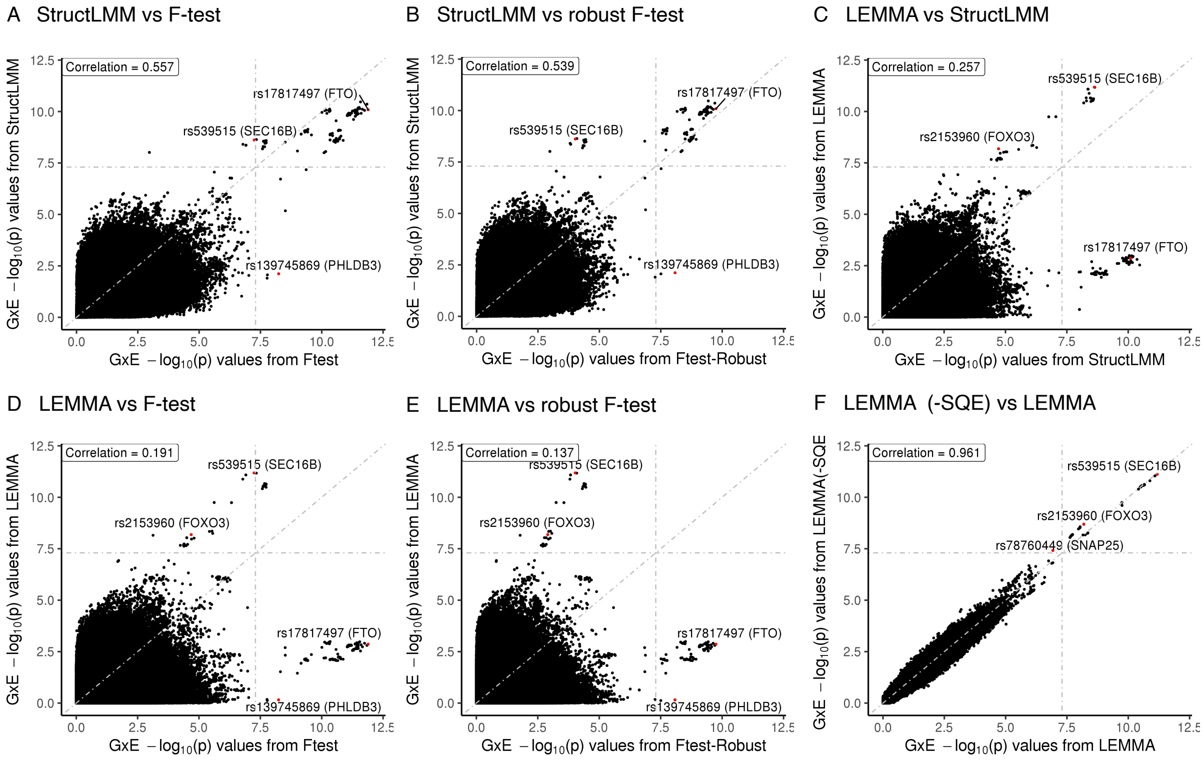

Figure S16: **Comparison of GxE association statistics for** $\log$**BMI.** Comparison of negative $\log_{10} p$ values obtained from LEMMA, StructLMM, the F-test and the robust F-test in an analysis of $\log$BMI in the UK Biobank. Grey lines denote ($p = 5 \times 10^{-8}$) and the $y = x$ axis. Pearson correlation is shown in a label at the top left of each plot. Red points denote the sentinel SNP for each locus.

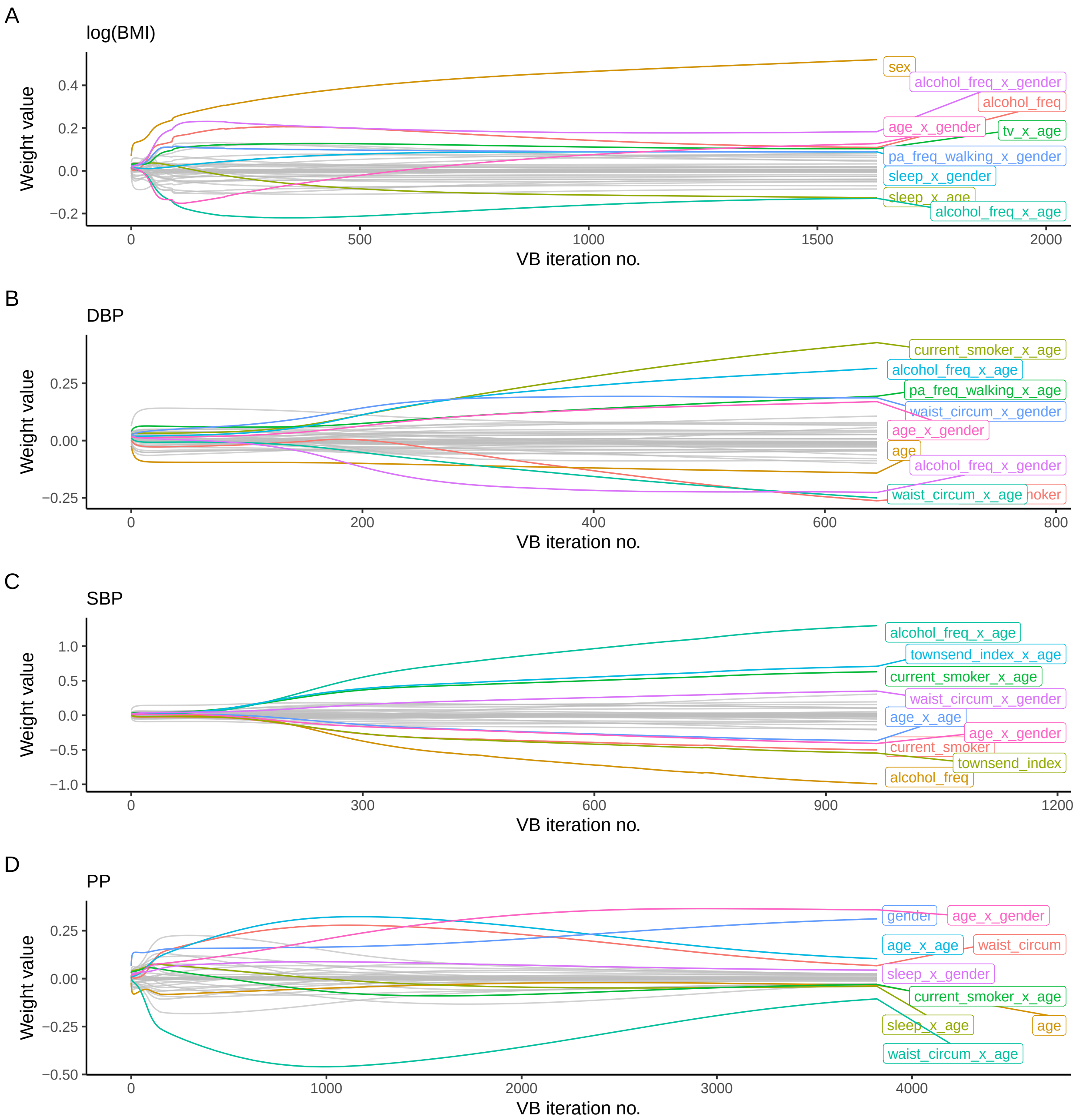

Figure S17: **Inference of environmental score weights from GxE analyses of four quantitative traits in the UK Biobank.** Evolution of the environmental score weights as LEMMA performs successive passes through the data.

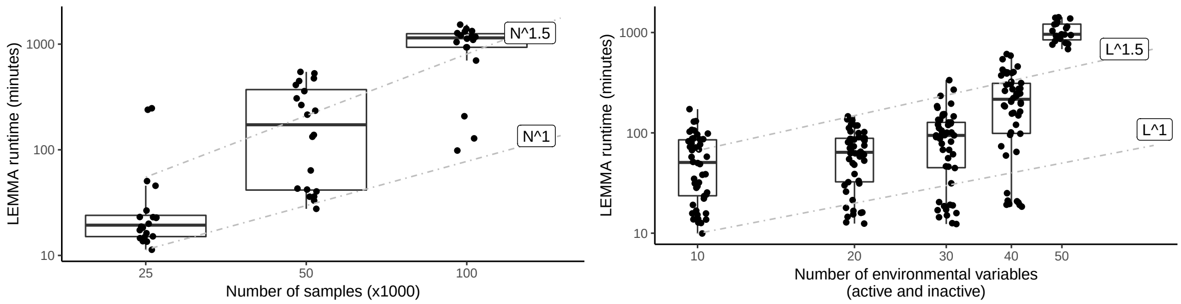

Figure S18: **Computational scaling** Log-log plots showing runtime of the variational bayes algorithm used to perform whole genome regression by LEMMA, as a function of sample size (left) and the number of environmental variables (right). Unless otherwise stated simulations were performed using $N = 25k$ samples, $M = 100k$ SNPs and $L = 30$ environmental variables. Phenotypes were constructed using 2500 non-zero main effects explaining 20% of variance, 1250 nonzero interaction effects explaining 5% of variance and 6 active environmental variables. See **Online methods** for full details of phenotype construction.

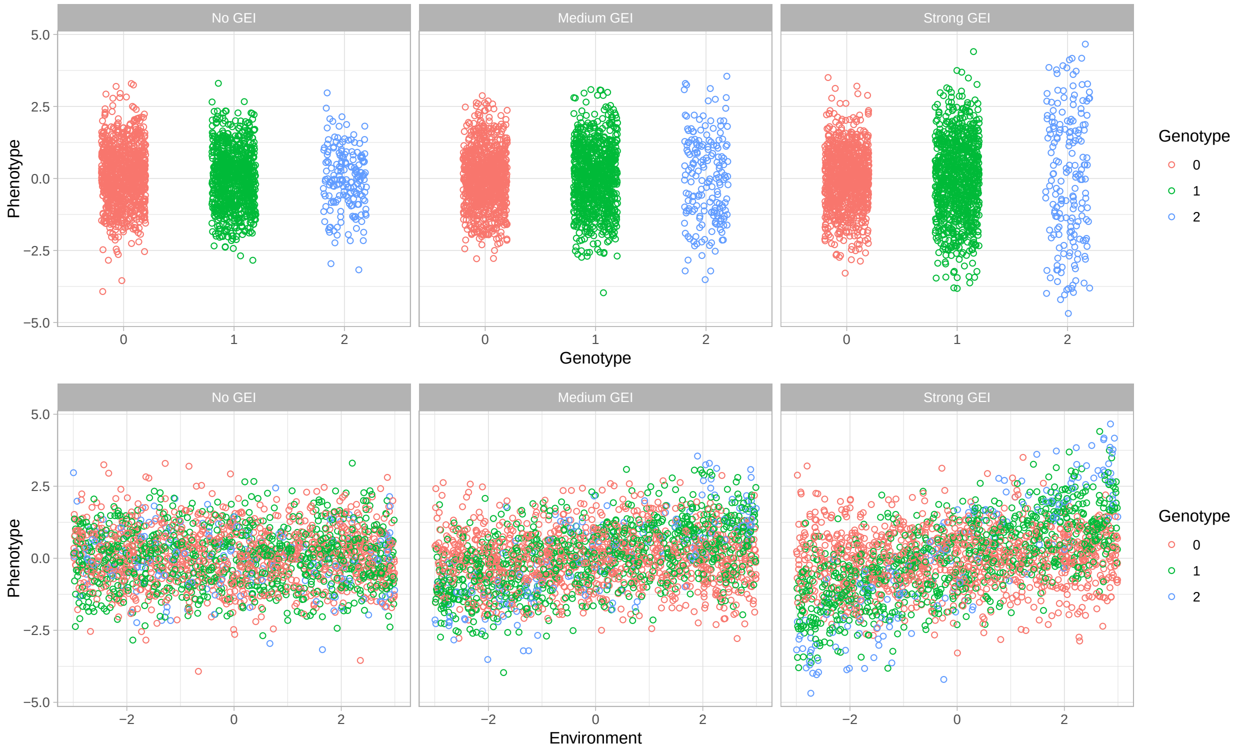

Figure S19: **Visualization of differences in variance induced by a multiplicative Gene-x-Environment effect.** Differences in phenotypic variance by genotype group (top) and by strength of the environmental exposure (bottom). The phenotype was simulated using 2000 individuals on the basis of a multiplication interaction between a single genotype (minor allele frequency 0.3) and an environment (uniformly distributed over $[-2, 2]$). From left to right; the GxE interaction explained $0\%, 20\%, 40\%$ of trait variance.

Trait	No. samples	No. SNPs	No. envs	No. envs-sq	No. other covars	No. covars total	Iterations for WGR converge	Time for WGR to converge*
log(BMI)	281149	642095	42	30	24	96	1631	78 hours 18 mins
PP	280749	642102	45	13	25	83	3821	183 hours 26 mins
SBP	280749	642102	45	15	25	85	967	46 hours 25 mins
DBP	280749	642102	45	15	25	85	646	31 hours 00 mins

Table S1: **Quality control and time to convergence of the WGR analyses**Time for the whole genome regression analysis to converge is reported for four quantitative traits in the UK Biobank, as well as the number of SNPs and samples passing quality control and the number of covariates controlled for. 'Other covariates' consisted of the top 20 genetic principal components as reported by the UK Biobank, age^3, $age^2 \times$ gender, $age^3 \times$ gender, a binary indicator for the genotype chip and (for blood pressure traits only) BMI. Environmental variables used (including lower orders of age and gender) are described in (**Online Methods**). To control for potential bias due to non-linear dependence between the phenotype and heritable environmental variables, we tested each environmental variable and included any significant squared effects as additional covariates (**Online Methods**) *based on the average per-iteration cost of 243 seconds, using 32 cores distributed across a cluster with Xeon E5-2667 v4 3.2Ghz processors.

Trait	Genotyped (SC)		Genotyped (LDMS)		Common Imputed (LDMS)	
	h^2_{G} (s.e)	h^2_{GxE} (s.e)	h^2_{G} (s.e)	h^2_{GxE} (s.e)	h^2_{G} (s.e)	h^2_{GxE} (s.e)
log BMI	0.259 (0.069)	0.071 (0.009)	0.237 (0.126)	0.086 (0.024)	0.274 (0.056)	0.093 (0.028)
PP	0.233 (0.039)	0.075 (0.018)	0.203 (0.084)	0.111 (0.021)	0.228 (0.051)	0.125 (0.028)
SBP	0.24 (0.053)	0.033 (0.003)	0.223 (0.095)	0.038 (0.017)	0.251 (0.05)	0.039 (0.023)
DBP	0.277 (0.034)	0.014 (0.001)	0.231 (0.079)	0.016 (0.017)	0.254 (0.05)	0.016 (0.02)

Table S2: **Partitioned heritability estimates for four quantitative traits in the UK Biobank**. Comparison of the heritability estimates obtained using genotyped SNPs with RHE-SC, genotyped SNPs with RHE-LDMS, and common imputed SNPs (MAF>0.1 in the full UK Biobank cohort) with RHE-LDMS. GxE heritability estimates were were obtained using the ES from each model fit. All analyses controlled for the same covariates used in the WGR analysis (including the top 20 principal components). Abbreviations; s.e, standard error estimated using the block jackknife (see **Online Methods**); h^2_{G}, heritability due to additive genetic effects; h^2_{GxE}, heritability due to multiplicative GxE effects; RHE, randomized HE-regression[18,19]; SC, single SNP component; LDMS, SNPs stratified by minor allele frequency and LDscore (20 components).

Method	No. signals	Genomic control (χ^2)	Genomic control (p-values)*
LEMMA	2	1.062	1.038
LEMMA-S	5	1.275	1.164
StructLMM (-SQE)	3	NA	1.236
F-test (-SQE)	4	NA	1.372
robust F-test (-SQE)	2	NA	1.034
LEMMA (-SQE)	3	1.065	1.04
LEMMA-S (-SQE)	6	1.288	1.171

Table S5: **Comparison of the number of genome-wide significant GxE associations and genomics control statistics from a GxE analysis of** $\log$**BMI in the UK Biobank** The number of independent loci (at least 0.5cM apart) with genome-wide significant GxE interaction effects and genomic control statistics for seven different methods applied to $\log$BMI in the UK Biobank. Genomic control is computed from GxE interaction tests statistics from $10,295,038$ imputed SNPs. Abbreviations; LEMMA-S, LEMMA with a homoskedastic test statistic (see **Online Methods**); (-SQE), significant squared environmental variables (Bonferroni correction) not included as additional covariates.

*The test statistics from StructLMM, F-test and the robust F-test are not χ^2_1 distributed. Hence for these methods we use $\lambda_{\text{GC}} = \log_{10}(m)/\log_1 0(0.5)$, where m is the median p-value, to denote the genomic control statistic as suggested by Moore *et al.*[20].

	logBMI ES	PP ES	SBP ES	DBP ES
PC1	2.182	0.023	0.467	0.403
PC2	0.254	0.153	0.197	0.088
PC3	0.022	0.382	0.405	0.295
PC4	0.657	0.465	0.091	0.095
PC5	15.075	2.652	68.293	71.439
PC6	0.703	0.780	0.020	0.480
PC7	0.469	0.095	0.933	0.998
PC8	0.778	1.898	2.784	1.848
PC9	0.675	0.878	14.130	27.305
PC10	0.814	0.081	0.647	0.276
PC11	2.759	0.255	6.547	7.970
PC12	0.319	0.974	0.486	0.511
PC13	0.659	0.508	0.475	2.003
PC14	3.301	5.779	2.575	4.454
PC15	0.186	0.418	0.300	0.531
PC16	3.554	3.141	2.830	6.560
PC17	0.695	1.590	1.208	0.061
PC18	3.965	0.215	0.681	0.884
PC19	0.350	0.375	0.099	0.409
PC20	2.463	1.208	1.228	0.720

Table S7: **Association between genetic principal components and the environmental score for four traits in the UK Biobank** Associations computed using ordinary least squares to regress the environmental score against the top 20 principal components (with an intercept included). Association strength reported using negative $\log_{10}(P)$-values from a standard t-test. Abbreviations; PC, genetic principal component; ES, environmental score.

Trait	Genotyped (LDMS)		Genotyped (LDMS), additionally controlling for ES-x-PCs	
	h^2_{G} (s.e)	h^2_{GxE} (s.e)	h^2_{G} (s.e)	h^2_{GxE} (s.e)
log BMI	0.2366 (0.1259)	0.0862 (0.0237)	0.2368 (0.1259)	0.0862 (0.0236)
PP	0.2025 (0.0841)	0.1110 (0.0208)	0.2025 (0.0840)	0.1113 (0.0208)
SBP	0.2225 (0.0954)	0.0377 (0.0168)	0.2226 (0.0955)	0.0360 (0.0166)
DBP	0.2308 (0.0793)	0.0157 (0.0166)	0.2308 (0.0793)	0.0142 (0.0163)

Table S8: **Sensitivity of partitioned heritability estimates to ES-x-PCs interaction in the UK Biobank**. Heritability estimates were computed using genotyped SNPs with RHE-LDMS and the ES from each WGR analysis. Left; heritability estimates obtained whilst controlling for the same covariates used in the WGR analysis (including the top 20 principal components), right; heritiability estimates obtained whilst additionally controlling for multiplicative interactios between the ES and genetic PCs. Abbreviations; s.e, standard error estimated using the block jackknife (see **Online Methods**); h^2_{G}, heritability due to additive genetic effects; h^2_{GxE}, heritability due to multiplicative GxE effects; RHE, randomized HE-regression[18,19]; LDMS, SNPs stratified by minor allele frequency and LDscore (20 components).

Trait	cor(ES,ES_{main})	abs(cor($X\beta$, $X\gamma$))	abs(cor(β, γ))
log(BMI)	-0.062	0.13680	0.05810
PP	-0.019	0.05306	0.03324
SBP	-0.297	0.01741	0.00816
DBP	-0.088	0.02464	0.00732

Table S9: **Correlation between main SNP effects and interaction SNP effects** Absolute correlation is used as γ is invariant to being multiplied by -1 (as LEMMA would apply the same transform to the ES).

1 Derivation of Variational Bayes updates

We use Coordinate Ascent Variational Inference (CAVI) to optimize the ELBO[1]. CAVI is a cyclic optimization strategy that iteratively maximizes the ELBO with respect to each latent variable whilst holding the others fixed. We now provide a brief justification of the CAVI update step and then derive the update for each of the latent variables in the LEMMA model.

Using the fact that the variational distributions factorizes, we can write the ELBO as

$$\begin{aligned}\mathcal{F}(\nu;\phi) &= \mathbb{E}_q\left[\log p(\theta, y|\mathcal{D}, \phi) - \log q(\theta)\right], \\ &= \log p(y|\mathcal{D},\phi) + \mathbb{E}_q\left[\log p(\theta|y,\mathcal{D},\phi)\right] - \sum_j \mathbb{E}_q\left[\log q(\theta_j)\right].\end{aligned}$$

Hence it is relatively simple to extract out dependance of $\mathcal{F}(\nu;\phi)$ on θ_j

$$\begin{aligned}\mathcal{F}_j &= \mathbb{E}_q\left[\log p(\theta_j|y,\theta_{-j},\mathcal{D},\phi)\right] - \mathbb{E}_q\left[\log q(\theta_j)\right] + \text{const}, \\ &= \int q(\theta_j)\left(\int \prod_{i\neq j} q(\theta_i)\log p(\theta_j|y,\theta_{-j},\mathcal{D},\phi)d\theta_{-j}\right)d\theta_j - \int q(\theta_j)\log q(\theta_j)d\theta_j + \text{const}, \\ &= \int q(\theta_j)\left(\mathbb{E}_{-\theta_j}\left[\log p(\theta_j|y,\theta_{-j},\mathcal{D},\phi)\right]\right)d\theta_j - \int q(\theta_j)\log q(\theta_j)d\theta_j + \text{const}.\end{aligned}$$

The last line is proportional to the KL divergence between $\log q(\theta_j)$ and $\mathbb{E}_{-\theta_j}\left[\log p(\theta|y,\phi)\right]$, where $\mathbb{E}_{-\theta_j}$ denotes the expectation with respect to the q distributions over all variables $\{\theta_i : \theta_i \neq \theta_j\}$. Therefore to maximize the ELBO with respect to $q(\theta_j)$ we must minimize the KL divergence between $\log q(\theta_j)$ and $\mathbb{E}_{-\theta_j}\left[\log p(\theta|y,\phi)\right]$, which occurs when

$$q^*(\theta_j) \propto \mathbb{E}_{-\theta_j}\left[\log p(\theta_j|y,\theta_{-j},\mathcal{D},\phi)\right].$$

After applying Bayes theorem, the above CAVI step can be equivalently expressed as

$$q^*(\theta_j) \propto \exp \mathbb{E}_{-\theta_j} \left[\log p(y|\theta_j, \theta_{-j}, \mathcal{D}, \phi) + \log p(\theta_j|\phi) \right]. \tag{1}$$

Updates for SNP main effect sizes $q(\beta_j)$ The prior and conditional log-likelihood for β_j are given by

$$p(\beta_j|\phi) = \lambda_\beta \mathcal{N}\left(\beta_j|0, \sigma_e^2 \sigma_{\beta,1}^2\right) + (1-\lambda_\beta)\mathcal{N}\left(\beta_j|0, \sigma_e^2\sigma_{\beta,2}^2\right), \tag{2}$$

$$\log p(y|\beta_j, \theta_{-\beta_j}, \mathcal{D}, \phi) = -\frac{1}{2\sigma_e^2}\left(\beta_j^2 ||X_j||_2^2 - 2\beta_j X_j^T y_{\text{resid},-\beta_j}\right) + \text{const}, \tag{3}$$

where const is a constant independent of β_j and $y_{\text{resid},-\beta_j} = y - C\alpha - X_{-j}\beta_{-j} - \eta \odot X\gamma$. Substituting eq. (3) and eq. (2) into eq. (1) yields

$$q^*(\theta_j) \propto \exp\left(-\frac{||X_j||_2^2}{2\sigma_e^2}\beta_j^2 + \frac{1}{\sigma_e^2} X_j^T \mathbb{E}_{-\beta_j}\left[y_{\text{resid},-\beta_j}\right]\beta_j\right) p(\beta_j|\phi) \tag{4}$$

as the prior is independent of $\theta_{-\beta_j}$. We now note the result

$$\exp\left(-\frac{||X_j||_2^2}{2\sigma_e^2}\beta_j^2 + \frac{1}{\sigma_e^2}X_j^T\mathbb{E}_{-\beta_j}\left[y_{\text{resid, -j}}\right]\beta_j\right)\mathcal{N}(\beta_j|0,\sigma_e^2\sigma_{\beta,i}^2) = \exp\left(\frac{\mu_{j,i}^2}{2s_{j,i}^\beta}\right)\sqrt{\frac{s_{j,i}^\beta}{\sigma_e^2\sigma_{\beta,i}^2}}\mathcal{N}(\beta_j|\mu_{j,i}^\beta, s_{j,i}^\beta), \tag{5}$$

where

$$s_{j,i}^\beta = \frac{\sigma_e^2}{||X_j||_2^2 + 1/\sigma_{\beta,i}^2}, \qquad \text{for } i = 1, 2$$

$$\mu_{j,i}^\beta = \frac{s_{j,i}^\beta}{\sigma_e^2} X_j^T \mathbb{E}_{-\beta_j}\left[y_{\text{resid},-\beta_j}\right], \qquad \text{for } i = 1, 2.$$

Substituting eq. (5) into eq. (4) yields

$$q^*(\beta_j) \propto \quad \lambda_\beta \exp\left(\frac{(\mu_{j,1}^\beta)^2}{2s_{j,1}^\beta}\right)\sqrt{\frac{s_{j,1}^\beta}{\sigma_e^2\sigma_{\beta,1}^2}}\mathcal{N}(\beta_j|\mu_{j,1}^\beta, s_{j,1}^\beta) + \tag{6}$$

$$(1-\lambda_\beta)\exp\left(\frac{(\mu_{j,2}^\beta)^2}{2s_{j,2}^\beta}\right)\sqrt{\frac{s_{j,2}^\beta}{\sigma_e^2\sigma_{\beta,2}^2}}\mathcal{N}(\beta_j|\mu_{j,2}^\beta, s_{j,2}^\beta). \tag{7}$$

It is now clear that $q^*(\beta_j)$ is the probability density function of a mixture of gaussians

$$q^*(\beta_j) = \psi_j^\beta \mathcal{N}(\beta_j|\mu_{j,1}^\beta, s_{j,1}^\beta) + (1-\psi_j^\beta)\mathcal{N}(\beta_j|\mu_{j,2}^\beta, s_{j,2}^\beta),$$

where the mixture components ψ_j^β and $(1-\psi_j^\beta)$ must sum to one. Therefore

$$\psi_j^\beta = \frac{\lambda_\beta \exp\left(\frac{(\mu_{j,1}^\beta)^2}{2s_{j,1}^\beta}\right)\sqrt{\frac{s_{j,1}^\beta}{\sigma_e^2\sigma_{\beta,1}^2}}}{\lambda_\beta \exp\left(\frac{(\mu_{j,1}^\beta)^2}{2s_{j,1}}\right)\sqrt{\frac{s_{j,1}^\beta}{\sigma_e^2\sigma_{\beta,1}^2}} + (1-\lambda_\beta)\exp\left(\frac{(\mu_{j,2}^\beta)^2}{2s_{j,2}^\beta}\right)\sqrt{\frac{s_{j,2}^\beta}{\sigma_e^2\sigma_{\beta,2}^2}}}$$

or equivalently

$$\psi_j^\beta = \text{sigmoid}\left(\text{logit}\left(\lambda_\beta\right) - \frac{1}{2}\log\left(\frac{\sigma_{\beta,1}^2 s_{j,2}^\beta}{s_{j,1}^\beta \sigma_{\beta,2}^2}\right) + \frac{(\mu_{j,1}^\beta)^2}{2s_{j,1}^\beta} - \frac{(\mu_{j,2}^\beta)^2}{2s_{j,2}^\beta}\right).$$

Therefore, the update equations for $q(\beta_j)$ can be summarised as

$$s_{j,i}^\beta = \frac{\sigma_e^2}{||X_j||_2^2 + 1/\sigma_{\beta,i}^2}, \qquad \text{for } i = 1,2$$

$$\mu_{j,i}^\beta = \frac{s_{j,i}^\beta}{\sigma_e^2} X_j^T \mathbb{E}_{-\beta_j}\left[y_{\text{resid},-\beta_j}\right], \qquad \text{for } i = 1,2$$

$$\psi_j^\beta = \text{sigmoid}\left(\text{logit}\left(\lambda_\beta\right) - \frac{1}{2}\log\left(\frac{\sigma_{\beta,1}^2 s_{j,2}^\beta}{s_{j,1}^\beta \sigma_{\beta,2}^2}\right) + \frac{(\mu_{j,1}^\beta)^2}{2s_{j,1}^\beta} - \frac{(\mu_{j,2}^\beta)^2}{2s_{j,2}^\beta}\right)$$

where

$$\mathbb{E}_{-\beta_j}\left[y_{\text{resid},-\beta_j}\right] = y - C\mathbb{E}_q\left[\alpha\right] - X_{-j}\mathbb{E}_q\left[\beta_{-j}\right] - \text{diag}\left(\mathbb{E}_q\left[\eta\right]\right) X\mathbb{E}_q\left[\gamma\right].$$

Updates for SNP interaction effect sizes $q(\gamma_j)$ The derivation of the variational update for $q(\gamma_j)$ is extremely similar to that of $q(\beta_j)$. The prior and conditional log-likelihood for γ_j are given by

$$p(\gamma_j|\phi) = \lambda_\gamma \mathcal{N}\left(\gamma_j|0, \sigma_e^2\sigma_{\gamma,1}^2\right) + (1-\lambda_\gamma)\mathcal{N}\left(\gamma_j|0, \sigma_e^2\sigma_{\gamma,2}^2\right), \tag{8}$$

$$\log p(y|\gamma_j, \theta_{-\gamma_j}, \mathcal{D}, \phi) = -\frac{1}{2\sigma_e^2}\left(\gamma_j^2||Z_j||_2^2 - 2\gamma_j Z_j^T y_{\text{resid},-\gamma_j}\right) + \text{const}, \tag{9}$$

where const is a constant independent of γ_j and $y_{\text{resid},-\gamma_j} = y - C\alpha - X\beta - \eta \odot X_{-j}\gamma_{-j}$. Substituting eq. (9) and eq. (8) into eq. (1) yields

$$q^*(\gamma_j) \propto \exp\left(-\frac{\gamma_j^2}{2\sigma_e^2}\mathbb{E}_{-\gamma_j}\left[||Z_j||_2^2\right] + \frac{1}{\sigma_e^2}\gamma_j X_j^T \mathbb{E}_{-\gamma_j}\left[\eta \odot y_{\text{resid},-\gamma_j}\right]\right) p(\gamma_j|\phi) \tag{10}$$

as the prior is independent of θ_{-j}. Following the same steps as used in the derivation of $q^*(\beta_j)$, is it clear that $q^*(\gamma_j)$ is also the probability density function of a mixture of gaussians

$$q^*(\gamma_j) = \psi_j^\gamma \mathcal{N}(\gamma_j|\mu_{j,1}^\gamma, s_{j,1}^\gamma) + (1-\psi_j^\gamma)\mathcal{N}(\gamma_j|\mu_{j,2}^\gamma, s_{j,2}^\gamma),$$

whose optimal CAVI updates are given by

$$\begin{aligned}
s_{j,i}^\gamma &= \frac{\sigma_e^2}{\mathbb{E}_{-\gamma_j}\left[||Z_j||_2^2\right] + 1/\sigma_{\gamma,i}^2}, && \text{for } i = 1,2 \\
\mu_{j,i}^\gamma &= \frac{s_{j,i}^\gamma}{\sigma_e^2} X_j^T \mathbb{E}_{-\gamma_j}\left[\eta \odot y_{\text{resid},-\gamma_j}\right], && \text{for } i = 1,2 \\
\psi_j^\gamma &= \text{sigmoid}\left(\text{logit}\left(\lambda_\gamma\right) - \frac{1}{2}\log\left(\frac{\sigma_{\gamma,1}^2 s_{j,2}^\gamma}{s_{j,1}^\gamma \sigma_{\gamma,2}^2}\right) + \frac{(\mu_{j,1}^\gamma)^2}{2s_{j,1}^\gamma} - \frac{(\mu_{j,2}^\gamma)^2}{2s_{j,2}^\gamma}\right)
\end{aligned}$$

where

$$\begin{aligned}
\mathbb{E}_{-\gamma_j}\left[y_{\text{resid},-\gamma_j}\right] &= y - C\mathbb{E}_q[\alpha] - X\mathbb{E}_q[\beta] - \text{diag}\left(\mathbb{E}_q[\eta]\right) X_{-j}\mathbb{E}_q[\gamma_{-j}], \\
\mathbb{E}_{-\gamma_j}\left[||Z_j||_2^2\right] &= X_{.j}^T \text{diag}\left(\mathbb{E}_q\left[\eta^2\right]\right) X_{.j}, \\
&= \sum_{l,m} \mathbb{E}_q[w_m]\,\mathbb{E}_q[w_l] \underbrace{\sum_i X_{ij}^2 E_{il} E_{im}}_{\text{precomputed}} \\
&\quad + \sum_l \text{Var}_q(w_l) \underbrace{\sum_i X_{ij}^2 E_{il}^2}_{\text{precomputed}}.
\end{aligned}$$

Note that computation of $\mathbb{E}_{-\gamma_j}\left[||Z_j||_2^2\right]$ is an $O(L^2 + N)$ operation due to the precomputation of $\sum_i X_{ij}^2 E_{il} E_{im}$ (and without this precomputation the compute cost of this update would be $\mathcal{O}(NL^2)$).

Updates for interaction weights $q(w_l)$ Rewriting the conditional log-likelihood makes its dependence on w clear

$$\begin{aligned}\log p(y|w,\theta_{-w},\mathcal{D},\phi) &= -\frac{1}{2\sigma_e^2}||y - C\alpha - X\beta - \eta\odot X\gamma||_2^2 + \text{const},\\ &= -\frac{1}{2\sigma_e^2}||y - C\alpha - X\beta - Bw||_2^2 + \text{const}\end{aligned}$$

where $B = \text{diag}\left(X\gamma\right)E$ and const is a constant independent of w. For convenience we denote the l'th column of B as B_l. Therefore the prior and conditional log-likelihood of w_l are given by

$$p(w_l) = \mathcal{N}\left(w_l|0,1\right), \tag{11}$$

$$\log p(y|w_l,\theta_{-w_l},\mathcal{D},\phi) = -\frac{1}{2\sigma_e^2}\left(w_l^2||B_l||_2^2 - 2w_l B_l^T y_{\text{resid},-w_l}\right) + \text{const}, \tag{12}$$

where $y_{\text{resid},-w_l} = y - C\alpha - X\beta - B_{-l}w_{-l}$ and const is now a constant independent of w_l. Substituting eq. (12) and eq. (11) into eq. (1) yields

$$\begin{aligned}q^*(w_l) &\propto \exp\left(-\frac{w_l^2}{2}\mathbb{E}_{-w_l}\left[||B_l||_2^2\right] + w_l\mathbb{E}_{-w_l}\left[B_l^T y_{\text{resid},-w_l}\right]\right)p(w_l),\\ &\propto \mathcal{N}(w_l|\mu_l^w, s_l^w),\end{aligned}$$

where

$$\begin{aligned}s_l^w &= \frac{\sigma_e^2}{\sigma_e^2 + \mathbb{E}_{-w_l}\left[||B_l||_2^2\right]},\\ \mu_l^w &= \frac{s_l^w}{\sigma_e^2}\mathbb{E}_{-w_l}\left[B_l^T y_{\text{resid},-w_l}\right].\end{aligned}$$

As $q^*(w_l)$ must be a valid distribution, it is clear that $q^*(w_l) = \mathcal{N}(w_l|\mu_l^w, s_l^w)$. The quantities $\mathbb{E}_{-w_l}\left[||B_l||_2^2\right]$ and $\mathbb{E}_{-w_l}\left[B_l^T y_{\text{resid},-w_l}\right]$ can be computed as follows

$$\begin{aligned}
\mathbb{E}_{-w_l}\left[||B_l||_2^2\right] &= \mathbb{E}\left[\sum_i E_{il}^2 \left(\sum_j X_{ij}^2 \gamma_j\right)^2\right], \\
&= \sum_i E_{il}^2 \left(\sum_j X_{ij}^2 \mathbb{E}\left[\gamma_j\right]\right)^2 + \sum_i E_{il}^2 \sum_j X_{ij}^2 \text{Var}\left(\gamma_j\right), \\
&= y_X^T \text{diag}\left(E_l^2\right) y_X + \sum_j \text{Var}\left(\gamma_j\right) \underbrace{\sum_i E_{il}^2 X_{ij}^2}_{\text{precomputed}}.
\end{aligned}$$

$$\begin{aligned}
\mathbb{E}_{-w_l}\left[B_l^T y_{\text{resid},-w_l}\right] = &\,(y - \hat{y}_M)^T \text{diag}\left(E_l^*\right) \hat{y}_X - E_l^* \text{diag}\left(\hat{y}_X^2\right) \mathbb{E}\left[\eta_{-l}\right] \\
&- \sum_j \text{Var}\left(\gamma_j\right) \sum_{m \neq l} \mathbb{E}\left[w_m\right] \underbrace{\sum_i X_{ij}^2 E_{il} E_{im}}_{\text{precomputed}}.
\end{aligned}$$

Note that computation of $\mathbb{E}_{-w_l}\left[B_l^T y_{\text{resid},-w_l}\right]$ is an $O(NL)$ operation due to the precomputation of $\sum_i X_{ij}^2 E_{il} E_{im}$ (and without this precomputation the compute cost of this update would be $\mathcal{O}(NML)$).

Updates for covariate main effect sizes $q(\alpha_c)$ The derivation of the variational update for $q(\alpha_c)$ is extremely similar to that of $q(w_l)$. The prior and conditional log-likelihood for α_c are given by

$$p(\alpha_c|\phi) = \mathcal{N}\left(\alpha_c|0, \sigma_e^2 \sigma_\alpha^2\right), \tag{13}$$

$$\log p(y|\alpha_c, \theta_{-\alpha_c}, \mathcal{D}, \phi) = -\frac{1}{2\sigma_e^2}\left(\alpha_c^2 ||C_c||_2^2 - 2\alpha_c C_c^T y_{\text{resid},-\alpha_c}\right) + \text{const}, \tag{14}$$

where const is a constant independent of α_c and $y_{\text{resid},-\alpha_c} = y - C_{-c}\alpha_{-c} - X\beta - \eta \odot X\gamma$. By similarity with the derivation of $q^*(w_l)$ it is clear that $q^*(\alpha_c)$ is a gassian distribution, with variational

updates

$$
\begin{aligned}
s_c^\alpha &= \frac{\sigma_e^2}{1/\sigma_\alpha^2 + (N-1)}, \\
\mu_c^\alpha &= \frac{s_c^\alpha}{\sigma_e^2} C_c^T \mathbb{E}_{-\alpha_c}\left[y_{\text{resid},-\alpha_c}\right]
\end{aligned}
$$

where $\mathbb{E}_{-\alpha_c}\left[y_{\text{resid},-\alpha_c}\right] = y - C_{-c}\mathbb{E}_q\left[\alpha_{-c}\right] - X\mathbb{E}_q\left[\beta\right] - \mathbb{E}_q\left[\eta\right] \odot X\mathbb{E}_q\left[\gamma\right]$.

2 Evidence lower bound

Variational inference involves maximising the evidence lower bound (ELBO) $\mathcal{F}(\phi;\nu)$ on the model log-likelihood $\log p(y|\mathcal{D},\phi)$. The ELBO can be separated into the expected conditional log-likelihood and the KL divergence between the variational distribution and the respective priors. This is given by

$$
\begin{aligned}
\mathcal{F}(\phi;\nu) &= \mathbb{E}_q\left[\log p(y|\theta,\mathcal{D},\phi)\right] - \sum_j \text{KL}(q(\theta_j;\nu_j)||p(\theta_j|\phi)), \\
&= -\frac{N}{2}\log(2\pi\sigma_e^2) \\
&\quad - \frac{1}{2\sigma_e^2}\left(||y - C\mathbb{E}_q\left[\alpha\right] - X\mathbb{E}_q\left[\beta\right] - \mathbb{E}_q\left[\eta\right] \odot X\mathbb{E}_q\left[\gamma\right]||_2^2\right) \\
&\quad - \frac{1}{2\sigma_e^2}\left(\mathbb{E}_q\left[\gamma\right]^T X^T \textbf{diag}\left(\mathbb{E}_q\left[\eta^2\right]\right) X\mathbb{E}_q\left[\gamma\right] - ||\mathbb{E}_q\left[\eta\right] \odot X\mathbb{E}_q\left[\gamma\right]||_2^2\right) \\
&\quad - \frac{N-1}{2\sigma_e^2}\sum_l \text{Var}_q\left(\alpha_l\right) - \frac{N-1}{2\sigma_e^2}\sum_k \text{Var}_q\left(\beta_k\right) \\
&\quad - \sum_c^{L'} KL(q(\alpha_c)\|p(\alpha_c)) - \sum_l^{L} KL(q(w_l)\|p(w_l)) \\
&\quad - \sum_j^{M} KL(q(\beta_j)\|p(\beta_j)) - \sum_j^{M} KL(q(\gamma_j)\|p(\gamma_j))
\end{aligned}
$$

While the KL Divergence between two univariate gaussian distributions is a standard result, the KL Divergence between two mixtures of gaussians is not analytically tractable. However the matched bound approximation [2] can be used to provide an upper bound when both have the same number of components. Thus for two mixtures of gaussians given by

$$u \sim \lambda \mathcal{N}(0, \sigma_1^2) + (1-\lambda)\mathcal{N}(0, \sigma_2^2),$$

$$v \sim \psi \mathcal{N}(\mu_1, s_1) + (1-\psi)\mathcal{N}(\mu_2, s_2),$$

the matched bound on the KL divergence is given by

$$\begin{aligned} KL(v\|u) \leq & \psi \log\frac{\psi}{\lambda} + (1-\psi)\log\frac{1-\psi}{1-\lambda} \\ & -\frac{1}{2} + \frac{\psi}{2}\left(\frac{s_1+\mu_1^2}{2\sigma_1^2} - \log\left(\frac{s_1}{\sigma_1^2}\right)\right) + \frac{(1-\psi)}{2}\left(\frac{s_2+\mu_2^2}{2\sigma_2^2} - \log\left(\frac{s_2}{\sigma_2^2}\right)\right). \end{aligned}$$

Use of the matched bound approximation retains a valid variational algorithm, because it maintains the lower bound on the marginal log-likelihood[3].

$$\begin{aligned} KL(q(\alpha_c)\|p(\alpha_c)) =& -\frac{1}{2} + \frac{1}{2}\left(\frac{s_m^\alpha + (\mu_m^\alpha)^2}{\sigma_e^2\sigma_\alpha^2} - \log\left(\frac{s_m^\alpha}{\sigma_e^2\sigma_\alpha^2}\right)\right) \\ KL(q(w_l)\|p(w_l)) =& -\frac{1}{2} + \frac{1}{2}\left((s_l^w + (\mu_l^w)^2) - \log(s_l^w)\right) \\ KL(q(\beta_j)\|p(\beta_j)) =& \psi_j^\beta \log\frac{\psi_j^\beta}{\lambda_\beta} + (1-\psi_j^\beta)\log\frac{1-\psi_j^\beta}{1-\lambda_\beta} - \frac{1}{2} \\ & + \frac{\psi_j^\beta}{2}\left(\frac{s_{j,1}^\beta + (\mu_{j,1}^\beta)^2}{\sigma_e^2\sigma_{\beta,1}^2} - \log\frac{s_{j,1}^\beta}{\sigma_e^2\sigma_{\beta,1}^2}\right) + \frac{1-\psi_j^\beta}{2}\left(\frac{s_{j,2}^\beta + (\mu_{j,2}^\beta)^2}{\sigma_e^2\sigma_{\beta,2}^2} - \log\left(\frac{s_{j,2}^\beta}{\sigma_e^2\sigma_{\beta,2}^2}\right)\right) \\ KL(q(\gamma_j)\|p(\gamma_j)) =& \psi_j^\gamma \log\frac{\psi_j^\gamma}{\lambda_\gamma} + (1-\psi_j^\gamma)\log\frac{1-\psi_j^\gamma}{1-\lambda_\gamma} - \frac{1}{2} \\ & + \frac{\psi_j^\gamma}{2}\left(\frac{s_{j,1}^\gamma + (\mu_{j,1}^\gamma)^2}{\sigma_e^2\sigma_{\gamma,1}^2} - \log\left(\frac{s_{j,1}^\gamma}{\sigma_e^2\sigma_{\gamma,1}^2}\right)\right) + \frac{1-\psi_j^\gamma}{2}\left(\frac{s_{j,2}^\gamma + (\mu_{j,2}^\gamma)^2}{\sigma_e^2\sigma_{\gamma,2}^2} - \log\left(\frac{s_{j,2}^\gamma}{\sigma_e^2\sigma_{\gamma,2}^2}\right)\right) \end{aligned}$$

3 Derivation of hyperparameter maximization

For the maximization step we set $\phi = \hat{\phi}$ where $\nabla_{\phi} F(\phi; \nu) = 0$. For ease of notation we perform the following change of variables

$$\tilde{\sigma}^2_{\beta,1} = \sigma^2_e \sigma^2_{\beta,1} \quad \rightarrow \frac{\partial}{\partial \tilde{\sigma}^2_{\beta,1}} = \frac{1}{\sigma^2_e} \frac{\partial}{\partial \sigma^2_{\beta,1}},$$
$$\tilde{\sigma}^2_{\beta,2} = \sigma^2_e \sigma^2_{\beta,2} \quad \rightarrow \frac{\partial}{\partial \tilde{\sigma}^2_{\beta,2}} = \frac{1}{\sigma^2_e} \frac{\partial}{\partial \sigma^2_{\beta,2}},$$

$$\tilde{\sigma}^2_{\gamma,1} = \sigma^2_e \sigma^2_{\gamma,1} \quad \rightarrow \frac{\partial}{\partial \tilde{\sigma}^2_{\gamma,1}} = \frac{1}{\sigma^2_e} \frac{\partial}{\partial \sigma^2_{\gamma,1}},$$
$$\tilde{\sigma}^2_{\gamma,2} = \sigma^2_e \sigma^2_{\gamma,2} \quad \rightarrow \frac{\partial}{\partial \tilde{\sigma}^2_{\gamma,2}} = \frac{1}{\sigma^2_e} \frac{\partial}{\partial \sigma^2_{\gamma,2}}.$$

This makes the derivation easier as all the partial derivatives become decoupled. Partial derivatives with respect to each hyper-parameter are given by

$$\frac{\partial F}{\partial \lambda_\beta} = \sum_j \left(\frac{\psi_j^\beta}{\lambda_\beta} - \frac{(1-\psi_j^\beta)}{1-\lambda_\beta} \right),$$
$$\frac{\partial F}{\partial \lambda_\gamma} = \sum_j \left(\frac{\psi_j^\gamma}{\lambda_\gamma} - \frac{(1-\psi_j^\gamma)}{1-\lambda_\gamma} \right),$$

$$\frac{\partial F}{\partial \tilde{\sigma}_{\beta,1}^2} = \sum_j \frac{\psi_j^\beta}{2} \left(-\frac{1}{\tilde{\sigma}_{\beta,1}^2} + \frac{s_{j,1}^\beta + (\mu_{j,1}^\beta)^2}{(\tilde{\sigma}_{\beta,1}^2)^2} \right),$$
$$\frac{\partial F}{\partial \tilde{\sigma}_{\beta,2}^2} = \sum_j \frac{1-\psi_j^\beta}{2} \left(-\frac{1}{\tilde{\sigma}_{\beta,2}^2} + \frac{s_{j,2}^\beta + (\mu_{j,2}^\beta)^2}{(\tilde{\sigma}_{\beta,2}^2)^2} \right),$$

$$\frac{\partial F}{\partial \tilde{\sigma}_{\gamma,1}^2} = \sum_j \frac{\psi_j^\gamma}{2} \left(-\frac{1}{\tilde{\sigma}_{\gamma,1}^2} + \frac{s_{j,1}^\gamma + (\mu_{j,1}^\gamma)^2}{(\tilde{\sigma}_{\gamma,1}^2)^2} \right),$$
$$\frac{\partial F}{\partial \tilde{\sigma}_{\gamma,2}^2} = \sum_j \frac{1-\psi_j^\gamma}{2} \left(-\frac{1}{\tilde{\sigma}_{\gamma,2}^2} + \frac{s_{j,2}^\gamma + (\mu_{j,2}^\gamma)^2}{(\tilde{\sigma}_{\gamma,2}^2)^2} \right),$$

$$\frac{\partial F}{\partial \sigma_e^2} = -\frac{N}{2\sigma_e^2} + \frac{1}{2(\sigma_e^2)^2}\mathbb{E}_q\left[||y - C\alpha - X\beta - \text{diag}(\eta)X\gamma||_2^2 \right] - \frac{M}{2\sigma_e^2} + \frac{1}{2(\sigma_e^2)^2\sigma_\alpha^2} \sum_c \left(s_c^\alpha + (\mu_c^\alpha)^2 \right)$$

Hence the maximization steps are

$$\hat{\lambda}_\beta = \frac{1}{P}\sum_j \psi_j^\beta, \tag{15}$$

$$\hat{\lambda}_\gamma = \frac{1}{P}\sum_j \psi_j^\gamma, \tag{16}$$

$$\tag{17}$$

$$\hat{\sigma}^2_{\beta,1} = \frac{\sum_j \psi_j^\beta (s^\beta_{j,1} + (\mu^\beta_{j,1})^2)}{\hat{\sigma}^2_e \sum_j \psi_j^\beta}, \tag{18}$$

$$\hat{\sigma}^2_{\beta,2} = \frac{\sum_j (1-\psi_j^\beta)(s^\beta_{j,2} + (\mu^\beta_{j,2})^2)}{\hat{\sigma}^2_e \sum_j (1-\psi_j^\beta)}, \tag{19}$$

$$\tag{20}$$

$$\hat{\sigma}^2_{\gamma,1} = \frac{\sum_j \psi_j^\gamma (s^\gamma_{j,1} + (\mu^\gamma_{j,1})^2)}{\hat{\sigma}^2_e \sum_j \psi_j^\gamma}, \tag{21}$$

$$\hat{\sigma}^2_{\gamma,2} = \frac{\sum_j (1-\psi_j^\gamma)(s^\gamma_{j,2} + (\mu^\gamma_{j,2})^2)}{\hat{\sigma}^2_e \sum_j (1-\psi_j^\gamma)}, \tag{22}$$

$$\tag{23}$$

$$\hat{\sigma}^2 = \frac{\mathbb{E}_q\left[||y - C\alpha - X\beta - \text{diag}\,(\eta)\, X\gamma||_2^2\right] + \frac{1}{\sigma^2_\alpha}\sum_c (s^\alpha_c + (\mu^\alpha_c)^2)}{N+M}. \tag{24}$$

As an aside we note that one could use the maximized hyper-parameters (after convergence) to obtain a point estimate of $\text{Var}\,(\beta)$. However, by substituting in Equations (15) to (22) we can see that this is equivalent to $\sum_j \mathbb{E}_q\left[\beta_j^2\right]/M$.

$$\begin{aligned}
\text{Var}\,(\beta) &= \lambda_\beta \sigma^2_{\beta,1} + (1-\lambda_\beta)\sigma^2_{\beta,2}, \\
&\approx \hat{\lambda}_\beta \hat{\sigma}^2_{\beta,1} + (1-\hat{\lambda}_\beta)\hat{\sigma}^2_{\beta,2}, \\
&= \frac{1}{M}\sum_j \left(\psi_j^\beta (s^\beta_{j,1} + (\mu^\beta_{j,1})^2) + (1-\psi_j^\beta)(s^\beta_{j,2} + (\mu^\beta_{j,2})^2)\right), \\
&= \frac{1}{M}\sum_j \mathbb{E}_q\left[\beta_j^2\right].
\end{aligned}$$

As the mean field assumption tends to cause variational inference algorithms to underestimate the variance of latent variables [4], this is likely to produce an underestimate of $\mathrm{Var}(\beta)$. We can observe the same result for $\mathrm{Var}(\gamma)$ with an analogous argument.

4 Compressed genotype data

To reduce RAM usage, LEMMA stores a compressed version of the genotype matrix using NM bytes. To do this LEMMA splits the interval $[0, 2]$ into 2^8 segments and stores the index of the segment that each dosage falls into, as well as the mean and variance for each SNP. Then when operating on a SNP, LEMMA reconstructs the centered and scaled dosages for that SNP. This approach is similar to that used by the BGEN data format [5] and results in a small loss of accuracy, but is more flexible that assuming dosages are hardcoded to $\{0, 1, 2\}$.

5 Computational efficiency

Using mean field variational inference, estimation of the posterior means of the latent variables β, γ, w can be reduced to an iterative algorithm that cycles through the variables sequentially, updating each conditional on the values of the others. Taking the main effect of the j'th SNP as an example, the update scheme for β_j can be written heuristically as

$$\tilde{\beta}_j = X_j^T y_{\text{resid}}, \tag{25}$$

$$\hat{\beta}_j^t = \text{regularise}(\tilde{\beta}_j; \phi^t), \tag{26}$$

$$y_{\text{resid}} = y_{\text{resid}} - (\hat{\beta}_j^t - \hat{\beta}_j^{t-1}) X_j^T. \tag{27}$$

In Equation (25) we compute the correlation between the j SNP and the residual phenotype vector. In Equation (26) we compute the posterior mean of β_j which depends on the correlation with the residual phenotype, the prior on β_j and the current hyper-parameters Finally in Equation (27) we update the residual phenotype vector.

The majority of computational time is spent on the dot product in Equation (25) and updating the residual phenotype in Equation (27). Both are BLAS Level 1 operations, which implies that memory access is often the principal bottleneck rather than the number of cores available. It is possible to step up to BLAS Level 2 by updating a block of SNPs in parallel [6], however this is still a memory bound operation. Instead we use a parallel computing strategy suggested by [7] for use in genome wide regression, and subsequently used by [8], to compute the dot product and perform the residual update in parallel using OpenMPI. Briefly, we partition the samples such that blocks of rows of the phenotype y, genotypes X and environmental variables E are assigned to each core. For a given update step, each core calculates the dot product for the locally held block of samples and then shares the local dot product with the rest of the network. From this the dot product for the entire cohort can be reconstructed cheaply. After computing the posterior mean, each core then updates the residual phenotype for the block of samples stored locally. We observed that a distributed algorithm using OpenMPI was faster than the same algorithm using multi-threaded matrix-vector operations with the Intel MKL Library even on a single node with multiple cores. However using OpenMPI has the additional advantage of allowing users to utilize cores from across a cluster rather than being restricted to a single node. **Figure S18** shows LEMMA scales with increasing sample size.

6 Pre-computed quantities

To aid computational efficiency we pre-compute a $M \times L(L+1)$ matrix W where

$$W_{j,m \times L+l} = \sum_i X_{ij}^2 E_{il} E_{im}, \quad \text{for } 1 \leq l < m \leq L \text{ and } 1 \leq j \leq M,$$

and is used in the updates of $q(w_l)$ and $q(\gamma_j)$. LEMMA can compute this internally, incurring a one off cost of $\mathcal{O}(NML^2)$, or is able to read from a text file at run time. As this is easily computed in parallel over batches of variants and/or environment, we recommend that for biobank scale datasets users should pre-compute this quantity beforehand using a separate tool that we have provided.

7 Parameter Initialization

We start the variational mean estimates of $q(\beta)$ and $q(\gamma)$ at zero. To initialize mean estimates of the interaction weights $q(w)$ we have two options; the first of which is simply to use a uniform weighting over all environments. For the second we apply an F-Test independently at each SNP and use the learned coefficients from the test with the lowest p-value as the initial values of the interaction weights. We find that we often obtain similar results from both options, so for simplicity we use a uniform start point for our Biobank analyses. To initialize mean estimates of $q(\alpha)$ we use the least squares fit of C on y.

Inintial values of the hyperparameters are drawn randomly from the following distributions

$$
\begin{aligned}
h_\beta^2 &\sim \mathcal{U}(0, 0.5), \\
h_\gamma^2 &\sim \mathcal{U}(0, 0.1), \\
-\log_{10}(\lambda_\beta) &\sim \mathcal{U}([2, ..., 1 - \log_{10}(M)]) \\
-\log_{10}(\lambda_\gamma) &\sim \mathcal{U}([2, ..., 1 - \log_{10}(M)]).
\end{aligned}
$$

We then set

$$
\begin{aligned}
\sigma_e^2 &= 1 - h_\beta^2 - h_\gamma^2, \\
\sigma_{\beta,1}^2 &= \frac{1}{\lambda_\beta M} \frac{h_\beta^2}{1 - h_\beta^2 - h_\gamma^2}, \\
\sigma_{\gamma,1}^2 &= \frac{1}{\lambda_\gamma M} \frac{h_\gamma^2}{1 - h_\beta^2 - h_\gamma^2},
\end{aligned}
$$

and initialize the spike variances at

$$
\begin{aligned}
\sigma_{\beta,2}^2 &= \sigma_{\beta,1}^2 / 1000, \\
\sigma_{\gamma,2}^2 &= \sigma_{\gamma,1}^2 / 1000.
\end{aligned}
$$

Setting the sparsity hyperparameters $\lambda_\beta, \lambda_\gamma$ in this manner allows LEMMA to start from a state where only a small number (somewhere between ten and one in one hundred) SNPs are expected to be part of the slab prior. The sparsity hyperparameters can then be updated in the variational maximization step to better reflect trait genetic architecture.

8 Missing data

Samples with missing data in the phenotype, environmental variables or covariates are excluded. By default LEMMA imputes missing genetic data with the mean dosage of each SNP, however as LEMMA does not assume dosages are hard called with $\{0, 1, 2\}$ we recommend that users first impute genetic data with standard imputation pipelines.

9 Robust standard errors in GxE Studies

In **Figure S19** we illustrate how a multiplicative GxE interaction effect on a quantitative trait can cause the conditional trait variance given an interacting SNP $\mathrm{Var}(Y|g_0)$ to differ according to the interacting SNPs genotype. This is known as conditional heteroskedasticity and is the key insight behind several recent methods to detect SNPs with non-zero GxE effects in the UK Biobank [9,10].

In the same figure, we can observe that the conditional trait variance given the environmental exposure $\mathrm{Var}(y|E)$ also displays signs of conditional heteroskedasticity. Previous studies [11] have observed that methods that assume heteroskedasticity can display substantial inflation when testing for GxE effects at SNPs where there is no true GxE effect. In our simulations we observed that inflation of GxE tests statistics from LEMMA-S and the F-test, both of which assume homoskedasticity, increased with SNP-GxE heritability. Below we give an explanation for this phenomenon.

Consider a polygenic quantitative trait Y that has multiplicative GxE interactions with the

same environmental exposure E at multiple SNPs

$$y_i = \alpha E_i + \sum_{j=1}^{M} \beta_j G_{ij} + \sum_{j=1}^{M} \gamma_j E_i G_{ij} + \epsilon_i, \tag{28}$$

where M is the number of SNPs and the coefficients represent true effects. For simplicity we assume that E and SNPs G_j are normalized to have mean zero and variance one, that the set of E with all causal SNPs $\{E\} \cup \{G_j : \beta_j \neq 0\}$ is pairwise independent and that the influence from population structure is negligible.

Suppose we have identified E as an environmental variable that may plausible have GxE interactions with our phenotype and we then conduct a GWAS for GxE effects. Then at the k'th SNP we wish to test the hypothesis $\gamma_k \neq 0$ in the following linear model

$$\begin{aligned} y &= \alpha E + G_k \beta_k + E \cdot G_k \gamma_k + u, \\ &= X\tau + u, \end{aligned}$$

where in the second line $\tau = (\alpha, \beta_k, \gamma_k)^T$, X is the corresponding design matrix encapsulating all fixed effects and u in an unobserved random effects capturing residual noise. Assuming that $\mathbb{E}\left[u|X\right] = 0$, the usual least squares estimate of τ, $\hat{\tau} = (X^T X)^{-1} X^T y$, has asymptotic distribution

$$\hat{\tau} \to \mathcal{N}(\tau, \mathrm{Var}\left(\hat{\tau}\right)),$$

where

$$\begin{aligned} \mathrm{Var}\left(\hat{\tau}\right) &= \mathbb{E}_X\left[\mathrm{Var}\left(\hat{\tau}|X\right)\right] + \mathrm{Var}_X\left(\mathbb{E}\left[\hat{\tau}|X\right]\right), \\ &= \mathbb{E}_X\left[\mathrm{Var}\left(\hat{\tau}|X\right)\right] + \mathrm{Var}_X\left(\tau\right), \\ &= \mathbb{E}_X\left[\mathrm{Var}\left(\hat{\tau}|X\right)\right], \end{aligned}$$

and

$$\begin{aligned}
\text{Var}\left(\hat{\tau}|X\right) &= \text{Var}\left(\tau + (X^TX)^{-1}X^Tu|X\right), \\
&= (X^TX)^{-1}\text{Var}\left(X^Tu|X\right)(X^TX)^{-T}, \\
&= (X^TX)^{-1}X^T\text{Var}\left(u|X\right)X(X^TX)^{-T}.
\end{aligned}$$

The usual approach is to assume that $\text{Var}\left(u|X\right) = \sigma^2 I$ (ie homoskedasticity), which yields the standard variance estimator $\text{Var}\left(\hat{\tau}|X\right) = \sigma^2(X^TX)^{-1}$. However, given the true generative model for y given in Equation (28), we can write u as

$$u = \sum_{j\neq k}\left(G_j\beta_j + EG_j\gamma_j\right) + \epsilon. \tag{29}$$

Therefore the conditional variance of u given X is given by

$$\begin{aligned}
\text{Var}\left(u|X\right) &= \text{Var}\left(u|E=e, G_k = g_k\right), \\
&= \text{Var}\left(\sum_{j\neq k}\left(G_j\beta_j + eG_j\gamma_j\right) + \epsilon\right), \\
&= \sum_{j\neq k}\text{Var}\left(\beta_jG_j\right) + \sum_{j\neq k}\text{Var}\left(\gamma_jeG_j\right) + 2\sum_{j\neq k}\text{Cov}\left(\beta_jG_j, \gamma_jeG_j\right) + 1 \\
&\quad + \sum_{j\neq k, m\neq k}\text{Cov}\left(\beta_jG_j, \beta_mG_m\right) + \sum_{j\neq k, m\neq k}\text{Cov}\left(\gamma_jeG_j, \gamma_meG_m\right) \\
&= \sum_{j\neq k}\left(\beta_j + e\gamma_j\right)^2 + 1,
\end{aligned}$$

where the covariances in the second line are all zero due to pairwise independence of the set $\{E\} \cup \{G_j : \beta_j \neq 0\}$. Thus the conditional trait variance will vary depending on the strength of environmental exposure either if there are a few SNPs with GxE interactions of large effect or if there are many SNPs with small yet non-zero interaction effects, and in either case homoskedasticity is unlikely to be an appropriate assumption.

Robust standard errors, alternatively called Huber-White, sandwich or "heteroskedastic-consistent" errors [12,13], are standard tools used in economics [14] to overcome this issue and have previously been proposed for use in GxE interaction studies [11,15,16]. We further include a small adjustment that reduces bias in small samples [17]. This yields the variance estimator

$$\mathrm{Var}\,(\hat{\tau}) = (H^T H)^{-1} H^T \hat{\Sigma} H (H^T H)^{-1},$$

where $\hat{\Sigma}$ is a diagonal matrix with $\hat{\Sigma}_{ii} = \frac{\hat{\epsilon}_i^2}{(1-h_{ii})^2}$, where $\hat{\epsilon} = y - H\hat{\tau}$ and $h = H(H^T H)^{-1} H^T$.

References

1. Bishop, C. M. *Pattern Recognition and Machine Learning* (Springer-Verlag New York, 2006).

2. Hershey, J. R. & Olsen, P. A. Approximating the Kullback Leibler Divergence Between Gaussian Mixture Models. In *2007 IEEE International Conference on Acoustics, Speech and Signal Processing - ICASSP '07*, vol. 4, IV–317–IV–320 (2007).

3. Wang, C. & Blei, D. M. Variational inference in nonconjugate models. *Journal of Machine Learning Research* **14**, 1005–1031 (2013). 1209.4360.

4. Blei, D. M., Kucukelbir, A. & McAuliffe, J. D. Variational Inference: A Review for Statisticians. *Journal of the American Statistical Association* **112**, 859–877 (2017).

5. Band, G. & Marchini, J. Bgen: a binary file format for imputed genotype and haplotype data. *bioRxiv* (2018).

6. Loh, P. R. *et al.* Efficient Bayesian mixed-model analysis increases association power in large cohorts. *Nature Genetics* **47**, 284–290 (2015).

7. Fernando, R. L., Dekkers, J. C. & Garrick, D. J. A class of Bayesian methods to combine large numbers of genotyped and non-genotyped animals for whole-genome analyses. *Genetics Selection Evolution* **46**, 1–13 (2014).

8. Powell, J. E. *et al.* Signatures of negative selection in the genetic architecture of human complex traits. *Nature Genetics* **50**, 746–753 (2018).

9. Young, A. I., Wauthier, F. L. & Donnelly, P. Identifying loci affecting trait variability and detecting interactions in genome-wide association studies. *Nature Genetics* **50**, 1608—-1614 (2018).

10. Wang, H. *et al.* Genotype-by-environment interactions inferred from genetic effects on phenotypic variability in the uk biobank. *Science Advances* **5** (2019).

11. Almli, L. M. *et al.* Correcting systematic inflation in genetic association tests that consider interaction effects application to a genome-wide association study of posttraumatic stress disorder. *JAMA Psychiatry* **71**, 1392–1399 (2014).

12. Huber, P. J. *et al.* The behavior of maximum likelihood estimates under nonstandard conditions. In *Proceedings of the fifth Berkeley symposium on mathematical statistics and probability*, vol. 1, 221–233 (University of California Press, 1967).

13. White, H. *et al.* A heteroskedasticity-consistent covariance matrix estimator and a direct test for heteroskedasticity. *econometrica* **48**, 817–838 (1980).

14. Greene, W. H. *Econometric Analysis 5th edition* (Pearson Education India, 2003).

15. Tchetgen, E. J. T. & Kraft, P. On the robustness of tests of genetic associations incorporating gene-environment interaction when the environmental exposure is misspecified. *Epidemiology* **22**, 257–261 (2011).

16. Voorman, A., Lumley, T., McKnight, B. & Rice, K. Behavior of QQ-plots and Genomic Control in studies of gene-environment interaction. *PLoS ONE* **6** (2011).

17. Long, J. S. & Ervin, L. H. Using heteroscedasticity consistent standard errors in the linear regression model. *The American Statistician* **54**, 217–224 (2000).

18. Wu, Y. & Sankararaman, S. A scalable estimator of SNP heritability for biobank-scale data. *Bioinformatics* **34**, i187–i194 (2018).

19. Pazokitoroudi, A. *et al.* Scalable multi-component linear mixed models with application to SNP heritability estimation. *bioRxiv* 522003 (2019).

20. Moore, R. *et al.* A linear mixed model approach to study multivariate gene-environment interactions. *Nat Genet* 180–186 (2019).
